# Supplementary material for: The High-Elevation Peatlands of the Northern Andes, Colombia
Source: Plants (Basel). 2023 Feb 20;12(4):955. doi: 10.3390/plants12040955 (PMC9967791; doi:10.3390/plants12040955)
Supplement: Supplementary file 1 [file plants-12-00955-s001.zip › plants-2132075-supplementary.pdf]

plants

MDPI

Article

The High-Elevation Peatlands of the Northern Andes, Colombia

Juan C. Benavides [1,*], Dale H. Vitt [2] and David J. Cooper [3]

[1] Departamento de Ecología y Territorio, Pontificia Universidad Javeriana, Bogotá 110231, Colombia
[2] School of Biological Sciences, Plant Biology, Southern Illinois University, Carbondale, IL 62901-6509, USA
[3] Department of Forest and Rangeland Stewardship, Colorado State University, Fort Collins, CO 80523-1572, USA
* Correspondence: jubenavides@javeriana.edu.co

Supplementary material

Tables

Table S1. Location, habitat, and identification of plant specimens collected and deposited at the COL and SIU herbaria indicating the collection number that corresponds to the collector Juan C. Benavides (JCB #) from 121 Andean peatlands in the Colombian Andes.

PLOT	CODELONG	Site	Habitat	SPECIES	Date	Col Number
IGU3	IGU3_20	Boyaca, Chiquiza, Laguna de Iguaque. 5.6906 N, −73.4356 W, 3660 m.a.s.l.	Lake fen. Small pond (kettle pond) sedimented with peat	Lepidozia sp	1/19/10	5200
IGU2	IGU2_4	Boyaca, Chiquiza, Laguna de Iguaque. 5.688 N, −73.4362 W, 3589 m.a.s.l.	Lake fen. Peatland on lake shore, vegetation dominated by S. magellanicum	Campylopus arctocarpus	1/19/10	5201
IGU6	IGU6_6	Boyaca, Chiquiza, Laguna de Cazadero (North side). 5.6952 N, −73.4221 W, 3576 m.a.s.l.	Lake fen. Lake fen on lake shore, of about 1 ha	Sphagnum sancto-josephense	1/20/10	5202
IGU1	IGU1_6	Boyaca, Chiquiza, Laguna de Iguaque. 5.6881 N, −73.4355 W, 3589 m.a.s.l.	Lake fen. Peatland on lake shore, vegetation dominated by S. magellanicum and Carex	Arcytophyllum muticum	1/19/10	5203
IGU3	IGU3_X1	Boyaca, Chiquiza, Laguna de Iguaque. 5.6906 N, −73.4356 W, 3660 m.a.s.l.	Lake fen. Small pond (kettle pond) sedimented with peat		1/19/10	5204
IGU2	IGU2_19	Boyaca, Chiquiza, Laguna de Iguaque. 5.688 N, −73.4362 W, 3589 m.a.s.l.	Lake fen. Peatland on lake shore, vegetation dominated by S. magellanicum		1/19/10	5205
IGU8	IGU8_17	Boyaca, Chiquiza, Laguna del Monte. 5.7087 N, −73.4243 W, 3831 m.a.s.l.	Lake fen. Small peatland (<400 m2) at lake shore	Riccardia paramorum	1/20/10	5206

IGU12	IGU12_X2	Boyaca, Chiquiza, Laguna Ojo de Agua. 5.6909 N, −73.4285 W, 3595 m.a.s.l.	Lake fen. Peatland with large hummocks (>100 m2) on lake shore		1/21/10	5207
IGU3	IGU3_X3	Boyaca, Chiquiza, Laguna de Iguaque. 5.6906 N, −73.4356 W, 3660 m.a.s.l.	Lake fen. Small pond (kettle pond) sedimented with peat		1/19/10	5208
IGU1	IGU1_X4	Boyaca, Chiquiza, Laguna de Iguaque. 5.6881 N, −73.4355 W, 3589 m.a.s.l.	Lake fen. Peatland on lake shore, vegetation dominated by S. magellanicum and Carex		1/19/10	5209
IGU1	IGU1_X4	Boyaca, Chiquiza, Laguna de Iguaque. 5.6881 N, −73.4355 W, 3589 m.a.s.l.	Lake fen. Peatland on lake shore, vegetation dominated by S. magellanicum and Carex		1/19/10	5210
DUE1	DUE1_2	Valle del Cauca, Mpio Riofrio, Venecia, Paramo El Duende. 4.0699 N, −76.5058 W, 3502 m.a.s.l.	Slope fen. Fen on seeping slope with Espeletia	Sphagnum oxyphyllum	6/3/10	5211
DUE6	DUE6_6	Valle del Cauca, Mpio Riofrio, Venecia, Paramo El Duende. 4.0715 N, −76.5066 W, 3499 m.a.s.l.	Slope fen. Sedge-dominated slope fen	Campylopus andersonii	6/3/10	5212
DUE1	DUE1_8	Valle del Cauca, Mpio Riofrio, Venecia, Paramo El Duende. 4.0699 N, −76.5058 W, 3502 m.a.s.l.	Slope fen. Fen on seeping slope with Espeletia	Breutelia chrysea	6/3/10	5213
DUE8	DUE8_4	Valle del Cauca, Mpio Riofrio, Venecia, Paramo El Duende. 4.0716 N, −76.5051 W, 3478 m.a.s.l.	Slope fen. Sedge-dominated slope fen	Calliergon stramineum	6/3/10	5214
DUE8	DUE8_10	Valle del Cauca, Mpio Riofrio, Venecia, Paramo El Duende. 4.0716 N, −76.5051 W, 3478 m.a.s.l.	Slope fen. Sedge-dominated slope fen	Sphagnum cf falcatulum Besch.	6/3/10	5215

DUE6	DUE6_9	Valle del Cauca, Mpio Riofrio, Venecia, Paramo El Duende. 4.0715 N, −76.5066 W, 3499 m.a.s.l.	Slope fen. Sedge-dominated slope fen	Riccardia paramorum	6/3/10	5216
DUE1	DUE1_11	Valle del Cauca, Mpio Riofrio, Venecia, Paramo El Duende. 4.0699 N, −76.5058 W, 3502 m.a.s.l.	Slope fen. Fen on seeping slope with Espeletia	Kurzia capillaris	6/3/10	5217
DUE8	DUE8_6	Valle del Cauca, Mpio Riofrio, Venecia, Paramo El Duende. 4.0716 N, −76.5051 W, 3478 m.a.s.l.	Slope fen. Sedge-dominated slope fen	Bartramia sp	6/3/10	5218
DUE1	DUE1_10	Valle del Cauca, Mpio Riofrio, Venecia, Paramo El Duende. 4.0699 N, −76.5058 W, 3502 m.a.s.l.	Slope fen. Fen on seeping slope with Espeletia	Riccardia paramorum	6/3/10	5219
DUE2	DUE2_13	Valle del Cauca, Mpio Riofrio, Venecia, Paramo El Duende. 4.0689 N, −76.506 W, 3510 m.a.s.l.	Slope fen. Sedge-dominated slope fen	Campylopus arctocarpus	6/3/10	5220
DUE7	DUE7_2	Valle del Cauca, Mpio Riofrio, Venecia, Paramo El Duende. 4.0725 N, −76.5018 W, 3438 m.a.s.l.	Slope fen. Sedge-dominated slope fen	Agrostis sp3	6/3/10	5221
DUE1	DUE1_X1	Valle del Cauca, Mpio Riofrio, Venecia, Paramo El Duende. 4.0699 N, −76.5058 W, 3502 m.a.s.l.	Slope fen. Fen on seeping slope with Espeletia		6/3/10	5222
DUE1	DUE1_X2	Valle del Cauca, Mpio Riofrio, Venecia, Paramo El Duende. 4.0699 N, −76.5058 W, 3502 m.a.s.l.	Slope fen. Fen on seeping slope with Espeletia		6/3/10	5223
DUE1	DUE1_X3	Valle del Cauca, Mpio Riofrio, Venecia, Paramo El Duende. 4.0699 N, −76.5058 W, 3502 m.a.s.l.	Slope fen. Fen on seeping slope with Espeletia		6/3/10	5224

DUE1	DUE1_X4	Valle del Cauca, Mpio Riofrio, Venecia, Paramo El Duende. 4.0699 N, −76.5058 W, 3502 m.a.s.l.	Slope fen. Fen on seeping slope with Espeletia		6/3/10	5225
DUE1	DUE1_X5	Valle del Cauca, Mpio Riofrio, Venecia, Paramo El Duende. 4.0699 N, −76.5058 W, 3502 m.a.s.l.	Slope fen. Fen on seeping slope with Espeletia		6/3/10	5226
DUE1	DUE1_X6	Valle del Cauca, Mpio Riofrio, Venecia, Paramo El Duende. 4.0699 N, −76.5058 W, 3502 m.a.s.l.	Slope fen. Fen on seeping slope with Espeletia		6/3/10	5227
DUE1	DUE1_X7	Valle del Cauca, Mpio Riofrio, Venecia, Paramo El Duende. 4.0699 N, −76.5058 W, 3502 m.a.s.l.	Slope fen. Fen on seeping slope with Espeletia		6/3/10	5228
NEV4	NEV4_9	Caldas, Murillo, PNN Los Nevados, Distichia peatland in the trail to Muzu. Near Laguna Verde. 4.8113 N, −75.3432 W, 4433 m.a.s.l.	Plateau fen. Peatland in old lake system but now is raised	Anastrophyllum nigrescens	3/25/10	5229
NEV8	NEV8_11	Caldas, Murillo, PNN Los Nevados, Distichia peatland, heads of Alfombrales. 4.8593 N, −75.3424 W, 4485 m.a.s.l.	Stream fen. Glacier-fed peatland in heads of stream	Fossombronia peruviana	3/26/10	5230
NEV5	NEV5_8	Caldas, Murillo, PNN Los Nevados, Distichia peatland heads of Rio Recio, and Alfombrales. 4.8505 N, −75.3325 W, 4465 m.a.s.l.	Stream fen. Glacier-fed peatland in heads of stream	Calliergon stramineum	3/26/10	5231
NEV6	NEV6_11	Caldas, Murillo, PNN Los Nevados, Distichia peatland, water divide between Rio Recio and Alfombrales. 4.854 N, −75.3344 W, 4486 m.a.s.l.	Stream fen. Glacier-fed peatland in heads of stream	Warnstorfia exannulata	3/26/10	5232

NEV2	NEV2_19	Caldas, Murillo, PNN Los Nevados, Distichia peatland in the trail to Muzu. 4.8103 N, −75.3422 W, 4432 m.a.s.l.	Plateau fen. Peatland in old lake system but now is raised	Liverwort	3/25/10	5233
NEV9	NEV9_2	Caldas, Murillo, PNN Los Nevados, Distichia peatland, heads of Alfombrales. 4.8603 N, −75.3475 W, 4485 m.a.s.l.	Stream fen. Glacier-fed peatland in heads of stream	Riccardia paramorum	3/26/10	5234
NEV4	NEV4_7	Caldas, Murillo, PNN Los Nevados, Distichia peatland in the trail to Muzu. Near Laguna Verde. 4.8113 N, −75.3432 W, 4433 m.a.s.l.	Plateau fen. Peatland in old lake system but now is raised	Lophozia sp2	3/25/10	5235
NEV1	NEV1_4	Caldas, Murillo, PNN Los Nevados, Distichia peatland in the trail to Muzu. 4.8527 N, −75.3649 W, 4432 m.a.s.l.	Plateau fen. Peatland in old lake system but now is raised	Isotachis sp	3/25/10	5236
NEV2	NEV2_5	Caldas, Murillo, PNN Los Nevados, Distichia peatland in the trail to Muzu. 4.8103 N, −75.3422 W, 4432 m.a.s.l.	Plateau fen. Peatland in old lake system but now is raised	Telaranea nematodes	3/25/10	5237
NEV1	NEV1_6	Caldas, Murillo, PNN Los Nevados, Distichia peatland in the trail to Muzu. 4.8527 N, −75.3649 W, 4432 m.a.s.l.	Plateau fen. Peatland in old lake system but now is raised	Sphagnum sp nov	3/25/10	5238
NEV8	NEV8_17	Caldas, Murillo, PNN Los Nevados, Distichia peatland, heads of Alfombrales. 4.8593 N, −75.3424 W, 4485 m.a.s.l.	Stream fen. Glacier-fed peatland in heads of stream	Aneura sp	3/26/10	5239
NEV7	NEV7_61	Caldas, Murillo, PNN Los Nevados, Distichia peatland, heads of Alfombrales. 4.8563 N, −75.3383 W, 4495 m.a.s.l.	Stream fen. Glacier-fed peatland in heads of stream		3/26/10	5240

NEV6	NEV6_12	Caldas, Murillo, PNN Los Nevados, Distichia peatland, water divide between Rio Recio and Alfombrales. 4.854 N, −75.3344 W, 4486 m.a.s.l.	Stream fen. Glacier-fed peatland in heads of stream	Breutelia chrysea	3/26/10	5241
NEV1	NEV1_12	Caldas, Murillo, PNN Los Nevados, Distichia peatland in the trail to Muzu. 4.8527 N, −75.3649 W, 4432 m.a.s.l.	Plateau fen. Peatland in old lake system but now is raised	Riccardia paramorum	3/25/10	5242
NEV5	NEV5_3	Caldas, Murillo, PNN Los Nevados, Distichia peatland heads of Rio Recio, and Alfombrales. 4.8505 N, −75.3325 W, 4465 m.a.s.l.	Stream fen. Glacier -fed peatland in heads of stream	Calliergon stramineum	3/26/10	5243
NEV1	NEV1_8	Caldas, Murillo, PNN Los Nevados, Distichia peatland in the trail to Muzu. 4.8527 N, −75.3649 W, 4432 m.a.s.l.	Plateau fen. Peatland in old lake system but now is raised	Sphagnum sp nov	3/25/10	5244
NEV2	NEV2_18	Caldas, Murillo, PNN Los Nevados, Distichia peatland in the trail to Muzu. 4.8103 N, −75.3422 W, 4432 m.a.s.l.	Plateau fen. Peatland in old lake system but now is raised	Adelanthus pittieri	3/25/10	5245
NEV3	NEV3_15	Caldas, Murillo, PNN Los Nevados, Distichia peatland in the trail to Muzu. Near Laguna Verde. 4.8114 N, −75.3428 W, 4432 m.a.s.l.	Plateau fen. Peatland in old lake system but now is raised	Lobatiriccardia sp	3/25/10	5246
NEV5	NEV5_5	Caldas, Murillo, PNN Los Nevados, Distichia peatland heads of Rio Recio, and Alfombrales. 4.8505 N, −75.3325 W, 4465 m.a.s.l.	Stream fen. Glacier-fed peatland in heads of stream	Lophozia sp2	3/26/10	5247
NEV5	NEV5_4	Caldas, Murillo, PNN Los Nevados, Distichia peatland heads of Rio Recio,	Stream fen. Glacier-fed peatland in heads of stream	Lophozia sp	3/26/10	5248

		and Alfombrales. 4.8505 N, −75.3325 W, 4465 m.a.s.l.				
NEV2	NEV2_15	Caldas, Murillo, PNN Los Nevados, Distichia peatland in the trail to Muzu. 4.8103 N, −75.3422 W, 4432 m.a.s.l.	Plateau fen. Peatland in old lake system but now is raised	Aneura sp	3/25/10	5249
NEV1	NEV1_7	Caldas, Murillo, PNN Los Nevados, Distichia peatland in the trail to Muzu. 4.8527 N, −75.3649 W, 4432 m.a.s.l.	Plateau fen. Peatland in old lake system but now is raised	Riccardia paramorum	3/25/10	5250
NEV7	NEV7_8	Caldas, Murillo, PNN Los Nevados, Distichia peatland, heads of Alfombrales. 4.8563 N, −75.3383 W, 4495 m.a.s.l.	Stream fen. Glacier-fed peatland in heads of stream	Riccardia paramorum	3/26/10	5251
NEV7	NEV7_9	Caldas, Murillo, PNN Los Nevados, Distichia peatland, heads of Alfombrales. 4.8563 N, −75.3383 W, 4495 m.a.s.l.	Stream fen. Glacier-fed peatland in heads of stream	Isotachis sp	3/26/10	5252
NEV2	NEV2_16	Caldas, Murillo, PNN Los Nevados, Distichia peatland in the trail to Muzu. 4.8103 N, −75.3422 W, 4432 m.a.s.l.	Plateau fen. Peatland in old lake system but now is raised	Lophozia sp	3/25/10	5253
NEV3	NEV3_9	Caldas, Murillo, PNN Los Nevados, Distichia peatland in the trail to Muzu. Near Laguna Verde. 4.8114 N, −75.3428 W, 4432 m.a.s.l.	Plateau fen. Peatland in old lake system but now is raised	Isotachis sp	3/25/10	5254
NEV2	NEV2_6	Caldas, Murillo, PNN Los Nevados, Distichia peatland in the trail to Muzu. 4.8103 N, −75.3422 W, 4432 m.a.s.l.	Plateau fen. Peatland in old lake system but now is raised	Lophozia sp2	3/25/10	5255

NEV2	NEV2_17	Caldas, Murillo, PNN Los Nevados, Distichia peatland in the trail to Muzu. 4.8103 N, −75.3422 W, 4432 m.a.s.l.	Plateau fen. Peatland in old lake system but now is raised	Pleurozium schreberi	3/25/10	5256
NEV1	NEV1_5	Caldas, Murillo, PNN Los Nevados, Distichia peatland in the trail to Muzu. 4.8527 N, −75.3649 W, 4432 m.a.s.l.	Plateau fen. Peatland in old lake system but now is raised	Breutelia chrysea	3/25/10	5257
NEV5	NEV5_2	Caldas, Murillo, PNN Los Nevados, Distichia peatland heads of Rio Recio, and Alfombrales. 4.8505 N, −75.3325 W, 4465 m.a.s.l.	Stream fen. Glacier-fed peatland in heads of stream	Bryum argenteum	3/26/10	5258
NEV5	NEV5_X1	Caldas, Murillo, PNN Los Nevados, Distichia peatland heads of Rio Recio, and Alfombrales. 4.8505 N, −75.3325 W, 4465 m.a.s.l.	Stream fen. Glacier-fed peatland in heads of stream		3/26/10	5259
NEV1	NEV1_X2	Caldas, Murillo, PNN Los Nevados, Distichia peatland in the trail to Muzu. 4.8527 N, −75.3649 W, 4432 m.a.s.l.	Plateau fen. Peatland in old lake system but now is raised		3/25/10	5260
NEV1	NEV1_X3	Caldas, Murillo, PNN Los Nevados, Distichia peatland in the trail to Muzu. 4.8527 N, −75.3649 W, 4432 m.a.s.l.	Plateau fen. Peatland in old lake system but now is raised		3/25/10	5261
NEV1	NEV1_X4	Caldas, Murillo, PNN Los Nevados, Distichia peatland in the trail to Muzu. 4.8527 N, −75.3649 W, 4432 m.a.s.l.	Plateau fen. Peatland in old lake system but now is raised		3/25/10	5262
NEV1	NEV1_X5	Caldas, Murillo, PNN Los Nevados, Distichia peatland in the trail to Muzu. 4.8527 N, −75.3649 W, 4432 m.a.s.l.	Plateau fen. Peatland in old lake system but now is raised		3/25/10	5263
NEV6	NEV6_X6	Caldas, Murillo, PNN Los Nevados, Distichia peatland, water divide	Stream fen. Glacier-fed peatland in heads of stream		3/26/10	5264

		between Rio Recio and Alfombrales. 4.854 N, −75.3344 W, 4486 m.a.s.l.				
NEV1	NEV1_X7	Caldas, Murillo, PNN Los Nevados, Distichia peatland in the trail to Muzu. 4.8527 N, −75.3649 W, 4432 m.a.s.l.	Plateau fen. Peatland in old lake system but now is raised		3/25/10	5265
NEV1	NEV1_X8	Caldas, Murillo, PNN Los Nevados, Distichia peatland in the trail to Muzu. 4.8527 N, −75.3649 W, 4432 m.a.s.l.	Plateau fen. Peatland in old lake system but now is raised		3/25/10	5266
NEV1	NEV1_X9	Caldas, Murillo, PNN Los Nevados, Distichia peatland in the trail to Muzu. 4.8527 N, −75.3649 W, 4432 m.a.s.l.	Plateau fen. Peatland in old lake system but now is raised		3/25/10	5267
NEV1	NEV1_X10	Caldas, Murillo, PNN Los Nevados, Distichia peatland in the trail to Muzu. 4.8527 N, −75.3649 W, 4432 m.a.s.l.	Plateau fen. Peatland in old lake system but now is raised		3/25/10	5268
NEV1	NEV1_X11	Caldas, Murillo, PNN Los Nevados, Distichia peatland in the trail to Muzu. 4.8527 N, −75.3649 W, 4432 m.a.s.l.	Plateau fen. Peatland in old lake system but now is raised		3/25/10	5269
CHI1	CHI1_6	Cundinamarca, Fomeque, PNN Chingaza, Heads of "Rio Frio", Laguna de Chingaza. 4.501 N, −73.7531 W, 3333 m.a.s.l.	Stream fen. Peatland in backwater floodplain from Rio Frio margins	Sphagnum cf falcatulum Besch.	3/12/10	5270
SUM2	SUM2_11	Sumapaz. Road to Nazareth. Fen close to trout fishery at roadside. 4.2613 N, −74.1909 W, 3552 m.a.s.l.	Slope fen. Slope fen with a small stream (< 10 cm wide) running through	Arcytophyllum muticum	1/27/09	3951
SUM1	SUM1_X1	Sumapaz. Road to Nazareth. Fen close to trout fishery at roadside. 4.2594 N, −74.1882 W, 3526 m.a.s.l.	Slope fen. Slope fen with a small stream (< 10 cm wide) running through		1/27/09	5272

SUM3	SUM3_4	Sumapaz. Road to Nazareth. Fen close to trout fishery at roadside. 4.2759 N, −74.1969 W, 3569 m.a.s.l.	Slope fen.	Anastrophyllum nigrescens	1/28/09	3956
SUM3	SUM3_4	Sumapaz. Road to Nazareth. Fen close to trout fishery at roadside. 4.2759 N, −74.1969 W, 3569 m.a.s.l.	Slope fen.	Vascular plant	1/28/09	3956
SUM7	SUM7_7	Sumapaz. Road to Nazareth. Fen close to trout fishery at roadside. 4.2759 N, −74.1969 W, 3645 m.a.s.l.	Lake fen. Small glacier cirques with small lake, peatland developed from terrestrialization of the lake	Warnstorfia exannulata	1/30/09	5274
SUM5	SUM5_8	Sumapaz. Road to Nazareth. Fen close to trout fishery at roadside. 4.2759 N, −74.1969 W, 3415 m.a.s.l.	Slope fen.	Sphagnum sancto-josephense	1/29/09	3976
SUM5	SUM5_8	Sumapaz. Road to Nazareth. Fen close to trout fishery at roadside. 4.2759 N, −74.1969 W, 3415 m.a.s.l.	Slope fen.	Sphagnum oxyphyllum	1/29/09	3976
SUM4	SUM4_14	Sumapaz. Road to Nazareth. Fen close to trout fishery at roadside. 4.2759 N, −74.1969 W, 3667 m.a.s.l.	Slope fen.	Lycopodiella glaucescens	1/28/09	3973
SUM1	SUM1_X2	Sumapaz. Road to Nazareth. Fen close to trout fishery at roadside. 4.2594 N, −74.1882 W, 3526 m.a.s.l.	Slope fen. Slope fen with a small stream (< 10 cm wide) running through		1/27/09	5277
SUM5	SUM5_9	Sumapaz. Road to Nazareth. Fen close to trout fishery at roadside. 4.2759 N, −74.1969 W, 3415 m.a.s.l.	Slope fen.	Lophocolea sp2	1/29/09	5278
MA10	MA10_9	Mamapacha, lake “Los Patos”. 5.1876 N, −73.272 W, 3145 m.a.s.l.	Stream fen. Stream fen on spill of Lake los patos	Bryum laevigatum	2/22/09	3931
MA10	MA10_9	Mamapacha, lake “Los Patos”. 5.1876 N, −73.272 W, 3145 m.a.s.l.	Stream fen. Stream fen on spill of Lake los patos	Pleurozium schreberi	2/22/09	3931

MA10	MA10_9	Mamapacha, lake "Los Patos". 5.1876 N, −73.272 W, 3145 m.a.s.l.	Stream fen. Stream fen on spill of Lake los patos	Bryum laevigatum	2/22/09	4054
MA10	MA10_9	Mamapacha, lake "Los Patos". 5.1876 N, −73.272 W, 3145 m.a.s.l.	Stream fen. Stream fen on spill of Lake los patos	Pleurozium schreberi	2/22/09	4054
MA1	MA1_4	Mamapacha, lake "La Tarea". 5.1918 N, −73.2649 W, 3059 m.a.s.l.	Slope fen. Lake fen, 50 m W from lake. Lake water level was raised around 8 m 10 years ago with a dam.	Sphagnum sp	2/17/09	3853
MA1	MA1_4	Mamapacha, lake "La Tarea". 5.1918 N, −73.2649 W, 3059 m.a.s.l.	Slope fen. Lake fen, 50 m W from lake. Lake water level was raised around 8 m 10 years ago with a dam.	Sphagnum sp	2/17/09	3879
MA3	MA3_13	Mamapacha, lake "La Tarea". 5.19 N, −73.2639 W, 3064 m.a.s.l.	Lake fen. Lake fen, floating mat on lake anchored to rock outcrop	Polytrichum commune	2/18/09	5281
MA11	MA11_22	Mamapacha, 300 m from way to Lake Lajadilla. 5.1864 N, −73.2701 W, 3180 m.a.s.l.	Slope fen. Small fen at outlet of small watershed less than 5 ha	Isotachis sp	2/22/09	5282
MA8	MA8_X1	Mamapacha, Lake "Lajadilla". 5.1842 N, −73.2749 W, 3240 m.a.s.l.	Stream fen. Small fen near to Lajadilla lake, fen formed by ridge stopping the flow of water towards the lake		2/21/09	5283
MA1	MA1_X2	Mamapacha, lake "La Tarea". 5.1918 N, −73.2649 W, 3059 m.a.s.l.	Slope fen. Lake fen, 50 m W from lake. Lake water level was raised around 8 m 10 years ago with a dam.		2/17/09	5284
MA4	MA4_X3	Mamapacha, lake "La Tarea". 5.1895 N, −73.2639 W, 3064 m.a.s.l.	Lake fen. Lake fen, floating mat on lake anchored to rock outcrop		2/18/09	5285
MA4	MA4_X4	Mamapacha, lake "La Tarea". 5.1895 N, −73.2639 W, 3064 m.a.s.l.	Lake fen. Lake fen, floating mat on lake anchored to rock outcrop		2/18/09	5286
RAB2	RAB2_2	Boyaca, Raquira, Paramo Rabanal, Sitio Calicanto. 5.4291 N, −73.5709 W, 3408 m.a.s.l.	Lake fen. Large peatland complex next to artificial reservoir	Sphagnum magellanicum	2/6/10	5287

RAB1	RAB1_11	Boyaca, Raquira, Cuchilla pena El Bosque. 5.4022 N, −73.5962 W, 3431 m.a.s.l.	Slope fen. Small hummocks in slight incline towards stream	Lophozia sp	2/5/10	5288
RAB1	RAB1_7	Boyaca, Raquira, Cuchilla pena El Bosque. 5.4022 N, −73.5962 W, 3431 m.a.s.l.	Slope fen. Small hummocks in slight incline towards stream	Campylopus arctocarpus	2/5/10	5289
RAB1	RAB1_8	Boyaca, Raquira, Cuchilla pena El Bosque. 5.4022 N, −73.5962 W, 3431 m.a.s.l.	Slope fen. Small hummocks in slight incline towards stream	Marchantia berteroana	2/5/10	5290
RAB1	RAB1_X1	Boyaca, Raquira, Cuchilla pena El Bosque. 5.4022 N, −73.5962 W, 3431 m.a.s.l.	Slope fen. Small hummocks in slight incline towards stream		2/5/10	5291
RAB1	RAB1_X2	Boyaca, Raquira, Cuchilla pena El Bosque. 5.4022 N, −73.5962 W, 3431 m.a.s.l.	Slope fen. Small hummocks in slight incline towards stream		2/5/10	5292
RAB1	RAB1_X3	Boyaca, Raquira, Cuchilla pena El Bosque. 5.4022 N, −73.5962 W, 3431 m.a.s.l.	Slope fen. Small hummocks in slight incline towards stream		2/5/10	5293
RAB1	RAB1_X4	Boyaca, Raquira, Cuchilla pena El Bosque. 5.4022 N, −73.5962 W, 3431 m.a.s.l.	Slope fen. Small hummocks in slight incline towards stream		2/5/10	5294
RAB1	RAB1_X5	Boyaca, Raquira, Cuchilla pena El Bosque. 5.4022 N, −73.5962 W, 3431 m.a.s.l.	Slope fen. Small hummocks in slight incline towards stream		2/5/10	5295
RAB1	RAB1_X6	Boyaca, Raquira, Cuchilla pena El Bosque. 5.4022 N, −73.5962 W, 3431 m.a.s.l.	Slope fen. Small hummocks in slight incline towards stream		2/5/10	5296

RAB1	RAB1_X7	Boyaca, Raquira, Cuchilla pena El Bosque. 5.4022 N, −73.5962 W, 3431 m.a.s.l.	Slope fen. Small hummocks in slight incline towards stream		2/5/10	5297
RAB1	RAB1_X8	Boyaca, Raquira, Cuchilla pena El Bosque. 5.4022 N, −73.5962 W, 3431 m.a.s.l.	Slope fen. Small hummocks in slight incline towards stream		2/5/10	5298
PAM1	PAM1_8	Norte de Santander, Pamplona, Laguna del Seminarista. Small sedimented lake. 7.3891 N, −72.6713 W, 2674 m.a.s.l.	Lake fen. Very small peatland bottom of old lake with sedges	Polytrichum commune	12/25/09	5299
PAM2	PAM2_1	Norte de Santander, Pamplona, Laguna del Seminarista. Small sedimented lake. 7.4083 N, −72.6429 W, 2475 m.a.s.l.	Lake fen. Small peatland in old lake basin	Sphagnum cf falcatulum Besch.	12/27/09	5300
YAR1	YAR1_X1	Antioquia, Santa Rosa de Osos, Llanos del Cuiba. 6.7565 N, −75.5082 W, 2764 m.a.s.l.	Stream fen. Very small peatland Sphagnum-dominated		2/28/10	5301
YAR1	YAR1_1	Antioquia, Santa Rosa de Osos, Llanos del Cuiba. 6.7565 N, −75.5082 W, 2764 m.a.s.l.	Stream fen. Very small peatland Sphagnum-dominated	Sphagnum sancto-josephense	2/28/10	5302
YAR1	YAR1_1	Antioquia, Santa Rosa de Osos, Llanos del Cuiba. 6.7565 N, −75.5082 W, 2764 m.a.s.l.	Stream fen. Very small peatland Sphagnum-dominated	Sphagnum sancto-josephense	2/28/10	5303
YAR1	YAR1_X2	Antioquia, Santa Rosa de Osos, Llanos del Cuiba. 6.7565 N, −75.5082 W, 2764 m.a.s.l.	Stream fen. Very small peatland Sphagnum-dominated		2/28/10	5304
TOL2	TOL2_8	Tolima, Anzoategui. Tolima Volcano. 4.6775 N, −75.3052 W, 4012 m.a.s.l.	Stream fen. Raised between a stream fen and a slope fen	Pernettya prostrata	4/25/09	5305
TOL5	TOL5_5	Tolima, Anzoategui. Tolima Volcano. 4.6766 N, −75.3085 W, 4105 m.a.s.l.	Lake fen. Lake fen on small lake (<1 ha) spill	Anastrophyllum nigrescens	4/27/09	5306

TOL3	TOL3_1	Tolima, Anzoategui. Tolima Volcano. 4.676 N, −75.3038 W, 4046 m.a.s.l.	Lake fen. Lake fed by a mix of underground volcanic water and runoff	Campylopus arctocarpus	4/26/09	5307
TOL1	TOL1_4	Tolima, Anzoategui. Tolima Volcano. 4.6779 N, −75.3014 W, 4010 m.a.s.l.	Stream fen. Stream fen on volcanic-fed spring	Riccardia paramorum	4/25/09	5308
TOL1	TOL1_6	Tolima, Anzoategui. Tolima Volcano. 4.6779 N, −75.3014 W, 4010 m.a.s.l.	Stream fen. Stream fen on volcanic-fed spring	Campylium polygamum	4/25/09	5309
TOL4	TOL4_7	Tolima, Anzoategui. Tolima Volcano. 4.6751 N, −75.3039 W, 4062 m.a.s.l.	Lake fen. Lake fen with cushions formed from vascular plants, distichia dominates at the center closest to the water and sphagnum and sedges near the shore	Campylopus andersonii	4/26/09	5310
TOL4	TOL4_7	Tolima, Anzoategui. Tolima Volcano. 4.6751 N, −75.3039 W, 4062 m.a.s.l.	Lake fen. Lake fen with cushions formed from vascular plants, distichia dominates at the center closest to the water and sphagnum and sedges near the shore	Sphagnum magellanicum	4/26/09	5310
TOL4	TOL4_5	Tolima, Anzoategui. Tolima Volcano. 4.6751 N, −75.3039 W, 4062 m.a.s.l.	Lake fen. Lake fen with cushions formed from vascular plants, distichia dominates at the center closest to the water and sphagnum and sedges near the shore	Campylopus nivalis	4/26/09	5311
TOL4	TOL4_5	Tolima, Anzoategui. Tolima Volcano. 4.6751 N, −75.3039 W, 4062 m.a.s.l.	Lake fen. Lake fen with cushions formed from vascular plants, distichia dominates at the center closest to the water and sphagnum and sedges near the shore	Cephalozia sp	4/26/09	5311
TOL4	TOL4_5	Tolima, Anzoategui. Tolima Volcano. 4.6751 N, −75.3039 W, 4062 m.a.s.l.	Lake fen. Lake fen with cushions formed from vascular plants, distichia dominates at the center closest to the water and sphagnum and sedges near the shore	Riccardia paramorum	4/26/09	5311
TOL4	TOL4_8	Tolima, Anzoategui. Tolima Volcano. 4.6751 N, −75.3039 W, 4062 m.a.s.l.	Lake fen. Lake fen with cushions formed from vascular plants, distichia dominates	Distichia muscoides	4/26/09	5312

			at the center closest to the water and sphagnum and sedges near the shore			
TOL1	TOL1_5	Tolima, Anzoategui. Tolima Volcano. 4.6779 N, −75.3014 W, 4010 m.a.s.l.	Stream fen. Stream fen on volcanic-fed spring	Brachytecium austroglareosum	4/25/09	5313
TOL1	TOL1_3	Tolima, Anzoategui. Tolima Volcano. 4.6779 N, −75.3014 W, 4010 m.a.s.l.	Stream fen. Stream fen on volcanic-fed spring	Campylium polygamum	4/25/09	5314
TOL1	TOL1_3	Tolima, Anzoategui. Tolima Volcano. 4.6779 N, −75.3014 W, 4010 m.a.s.l.	Stream fen. Stream fen on volcanic-fed spring	Polytrichum commune	4/25/09	5314
TOL2	TOL2_3	Tolima, Anzoategui. Tolima Volcano. 4.6775 N, −75.3052 W, 4012 m.a.s.l.	Stream fen. Raised bog between a stream fen and a slope fen	Riccardia paramorum	4/25/09	5315
TOL2	TOL2_3	Tolima, Anzoategui. Tolima Volcano. 4.6775 N, −75.3052 W, 4012 m.a.s.l.	Stream fen. Raised bog between a stream fen and a slope fen	Sphagnum tenerum	4/25/09	5315
TOL2	TOL2_1	Tolima, Anzoategui. Tolima Volcano. 4.6775 N, −75.3052 W, 4012 m.a.s.l.	Stream fen. Raised bog between a stream fen and a slope fen	Sphagnum tenerum	4/25/09	5316
TOL1	TOL1_9	Tolima, Anzoategui. Tolima Volcano. 4.6779 N, −75.3014 W, 4010 m.a.s.l.	Stream fen. Stream fen on volcanic-fed spring	Bryum argenteum	4/25/09	5317
TOL1	TOL1_2	Tolima, Anzoategui. Tolima Volcano. 4.6779 N, −75.3014 W, 4010 m.a.s.l.	Stream fen. Stream fen on volcanic-fed spring	Marchantia berteroana	4/25/09	5318
TOL1	TOL1_X1	Tolima, Anzoategui. Tolima Volcano. 4.6779 N, −75.3014 W, 4010 m.a.s.l.	Stream fen. Stream fen on volcanic-fed spring		4/25/09	5319
TOL1	TOL1_X2	Tolima, Anzoategui. Tolima Volcano. 4.6779 N, −75.3014 W, 4010 m.a.s.l.	Stream fen. Stream fen on volcanic-fed spring		4/25/09	5320
TOL5	TOL5_X3	Tolima, Anzoategui. Tolima Volcano. 4.6766 N, −75.3085 W, 4105 m.a.s.l.	Lake fen. Lake fen on small lake (<1 ha) spill		4/27/09	5321
TOL1	TOL1_X4	Tolima, Anzoategui. Tolima Volcano. 4.6779 N, −75.3014 W, 4010 m.a.s.l.	Stream fen. Stream fen on volcanic-fed spring		4/25/09	5322
TOL2	TOL2_X5	Tolima, Anzoategui. Tolima Volcano. 4.6775 N, −75.3052 W, 4012 m.a.s.l.	Stream fen. Raised bog between a stream fen and a slope fen		4/25/09	5323

TOL1	TOL1_X6	Tolima, Anzoategui. Tolima Volcano. 4.6779 N, −75.3014 W, 4010 m.a.s.l.	Stream fen. Stream fen on volcanic-fed spring		4/25/09	5324
TOL2	TOL2_X7	Tolima, Anzoategui. Tolima Volcano. 4.6775 N, −75.3052 W, 4012 m.a.s.l.	Stream fen. Raised bog between a stream fen and a slope fen		4/25/09	5325
TOL1	TOL1_X8	Tolima, Anzoategui. Tolima Volcano. 4.6779 N, −75.3014 W, 4010 m.a.s.l.	Stream fen. Stream fen on volcanic-fed spring		4/25/09	5326
TOL1	TOL1_X10	Tolima, Anzoategui. Tolima Volcano. 4.6779 N, −75.3014 W, 4010 m.a.s.l.	Stream fen. Stream fen on volcanic-fed spring		4/25/09	5327
NEG1	NEG1_4	Caldas, Manizales, Laguna Negra at Entrance of PNN "Los Nevados". 4.9804 N, −75.3376 W, 3904 m.a.s.l.	Lake fen. Fen in outlet if disturbed lake with evidence of flashfloods from glacier melting and volcano activity	Sphagnum oxyphyllum	3/27/10	5328
NEG2	NEG2_1	Caldas, Manizales, Laguna Negra at Entrance of PNN "Los Nevados". 4.9802 N, −75.3376 W, 3904 m.a.s.l.	Lake fen. Fen in outlet if disturbed lake with evidence of flash floods from glacier melting and volcano activity	Ribes sp	3/27/10	5329
NEG3	NEG3_2	Caldas, Manizales, Laguna Negra at Entrance of PNN "Los Nevados". 4.9803 N, −75.3378 W, 3905 m.a.s.l.	Lake fen. Fen in outlet if disturbed lake with evidence of flash floods from glacier melting and volcano activity	Sphagnum magellanicum	3/27/10	5330
NEG3	NEG3_3	Caldas, Manizales, Laguna Negra at Entrance of PNN "Los Nevados". 4.9803 N, −75.3378 W, 3905 m.a.s.l.	Lake fen. Fen in outlet if disturbed lake with evidence of flash floods from glacier melting and volcano activity	Lophozia sp	3/27/10	5331
NEG3	NEG3_8	Caldas, Manizales, Laguna Negra at Entrance of PNN "Los Nevados". 4.9803 N, −75.3378 W, 3905 m.a.s.l.	Lake fen. Fen in outlet if disturbed lake with evidence of flash floods from glacier melting and volcano activity	Lophozia sp2	3/27/10	5332
LAVE1	LAVE1_13	Cundinamarca, Teusa, Paramo Laguna Verde, Lake eastward outlet. 5.2151 N, −74.0012 W, 3630 m.a.s.l.	Lake fen. Peatland in old lake basin that is now part of the outlet, with large hummocks of vascular plant	Sphagnum sancto-josephense	3/15/10	5333

LAVE1	LAVE1_14	Cundinamarca, Teusa, Paramo Laguna Verde, Lake eastward outlet. 5.2151 N, −74.0012 W, 3630 m.a.s.l.	Lake fen. Peatland in old lake basin that is now part of the outlet, with large hummocks of vascular plant	Isotachis sp	3/15/10	5334
LAVE1	LAVE1_15	Cundinamarca, Teusa, Paramo Laguna Verde, Lake eastward outlet. 5.2151 N, −74.0012 W, 3630 m.a.s.l.	Lake fen. Peatland in old lake basin that is now part of the outlet, with large hummocks of vascular plant	Riccardia paramorum	3/15/10	5335
LAVE3	LAVE3_10	Cundinamarca, Teusa, Paramo Laguna Verde, North shore. 5.2174 N, −73.9963 W, 3623 m.a.s.l.	Lake fen. Peatland in old lake basin that is now part of the outlet, with large hummocks of vascular plant	Bartramia sp	3/16/10	5336
BE11	BE11_1	Antioquia, Belmira, Paramo de Belmira, Sitio La Laguna. 6.6668 N, −75.6691 W, 3269 m.a.s.l.	Stream fen. Large raised peatland in upland plateau	Symphyogyna sp	2/24/10	5337
BE11	BE11_10	Antioquia, Belmira, Paramo de Belmira, Sitio La Laguna. 6.6668 N, −75.6691 W, 3269 m.a.s.l.	Stream fen. Large raised peatland in upland plateau	Sphagnum sancto-josephense	2/24/10	5338
BE11	BE11_11	Antioquia, Belmira, Paramo de Belmira, Sitio La Laguna. 6.6668 N, −75.6691 W, 3269 m.a.s.l.	Stream fen. Large raised peatland in upland plateau	Cephalozia sp	2/24/10	5339
BE11	BE11_13	Antioquia, Belmira, Paramo de Belmira, Sitio La Laguna. 6.6668 N, −75.6691 W, 3269 m.a.s.l.	Stream fen. Large raised peatland in upland plateau	Hypnum cupressiforme	2/24/10	5340
BE11	BE11_14	Antioquia, Belmira, Paramo de Belmira, Sitio La Laguna. 6.6668 N, −75.6691 W, 3269 m.a.s.l.	Stream fen. Large raised peatland in upland plateau	Sphagnum cf falcatulum Besch.	2/24/10	5341
BE11	BE11_15	Antioquia, Belmira, Paramo de Belmira, Sitio La Laguna. 6.6668 N, −75.6691 W, 3269 m.a.s.l.	Stream fen. Large raised peatland in upland plateau	Calypogeia sp	2/24/10	5342

BE11	BE11_16	Antioquia, Belmira, Paramo de Belmira, Sitio La Laguna. 6.6668 N, −75.6691 W, 3269 m.a.s.l.	Stream fen. Large raised peatland in upland plateau	Telaranea nematodes	2/24/10	5343
BE12	BE12_1	Antioquia, Belmira, Paramo de Belmira, Sitio La Laguna. 6.6719 N, −75.669 W, 3272 m.a.s.l.	Stream fen. Large raised peatland in upland plateau	Sphagnum cf falcatulum Besch.	2/24/10	5344
BE12	BE12_6	Antioquia, Belmira, Paramo de Belmira, Sitio La Laguna. 6.6719 N, −75.669 W, 3272 m.a.s.l.	Stream fen. Large raised peatland in upland plateau	Symphyogyna sp	2/24/10	5345
BE12	BE12_7	Antioquia, Belmira, Paramo de Belmira, Sitio La Laguna. 6.6719 N, −75.669 W, 3272 m.a.s.l.	Stream fen. Large raised peatland in upland plateau	Sphagnum oxyphyllum	2/24/10	5346
BE8	BE8_1	Antioquia, Belmira. 6.6974 N, −75.6691 W, 3017 m.a.s.l.	Slope fen. Fen in extremely disturbed peatland in abandoned pasture	Sphagnum sancto-josephense	3/8/09	5347
BE9	BE9_1	Antioquia, Belmira. 6.6974 N, −75.6691 W, 2989 m.a.s.l.	Slope fen. Fen in extremely disturbed peatland in abandoned pasture	Sphagnum sancto-josephense	3/8/09	5348
CHI1	CHI1_1	Cundinamarca, Fomeque, PNN Chingaza, Heads of “Rio Frio”, Laguna de Chingaza. 4.501 N, −73.7531 W, 3333 m.a.s.l.	Stream fen. Peatland in backwater floodplain from Rio Frio margins	Sphagnum cundinamarcanum	3/12/10	5349
CHI1	CHI1_3	Cundinamarca, Fomeque, PNN Chingaza, Heads of “Rio Frio”, Laguna de Chingaza. 4.501 N, −73.7531 W, 3333 m.a.s.l.	Stream fen. Peatland in backwater floodplain from Rio Frio margins	Hypnum cupressiforme	3/12/10	5350
CHI1	CHI1_4	Cundinamarca, Fomeque, PNN Chingaza, Heads of “Rio Frio”, Laguna de Chingaza. 4.501 N, −73.7531 W, 3333 m.a.s.l.	Stream fen. Peatland in backwater floodplain from Rio Frio margins	Breutelia chrysea	3/12/10	5351

CHI1	CHI1_7	Cundinamarca, Fomeque, PNN Chingaza, Heads of “Rio Frio”, Laguna de Chingaza. 4.501 N, −73.7531 W, 3333 m.a.s.l.	Stream fen. Peatland in backwater floodplain from Rio Frio margins	lachemilla orbicularis	3/12/10	5352
CHI2	CHI2_4	Cundinamarca, Fomeque, PNN Chingaza, Heads of “Rio Frio”, Laguna de Chingaza. 4.5026 N, −73.7523 W, 3328 m.a.s.l.	Stream fen. Peatland in backwater floodplain from Rio Frio margins	Drepanocladus aduncus	3/12/10	5353
CHI3	CHI3_1	Cundinamarca, Fomeque, PNN Chingaza, Peatland on margins of Laguna de Chingaza. 4.5214 N, −73.7496 W, 3180 m.a.s.l.	Lake fen. Peatland in old lake basin	Sphagnum sancto-josephense	3/13/10	5354
CHI3	CHI3_2	Cundinamarca, Fomeque, PNN Chingaza, Peatland on margins of Laguna de Chingaza. 4.5214 N, −73.7496 W, 3180 m.a.s.l.	Lake fen. Peatland in old lake basin	Polytrichum commune	3/13/10	5355
CHI3	CHI3_5	Cundinamarca, Fomeque, PNN Chingaza, Peatland on margins of Laguna de Chingaza. 4.5214 N, −73.7496 W, 3180 m.a.s.l.	Lake fen. Peatland in old lake basin	Drepanocladus aduncus	3/13/10	5356
CHI3	CHI3_8	Cundinamarca, Fomeque, PNN Chingaza, Peatland on margins of Laguna de Chingaza. 4.5214 N, −73.7496 W, 3180 m.a.s.l.	Lake fen. Peatland in old lake basin	Drepanocladus longifolius	3/13/10	5357
CHI3	CHI3_11	Cundinamarca, Fomeque, PNN Chingaza, Peatland on margins of Laguna de Chingaza. 4.5214 N, −73.7496 W, 3180 m.a.s.l.	Lake fen. Peatland in old lake basin	Brachytecium austroglareosum	3/13/10	5358

CHI3	CHI3_14	Cundinamarca, Fomeque, PNN Chingaza, Peatland on margins of Laguna de Chingaza. 4.5214 N, −73.7496 W, 3180 m.a.s.l.	Lake fen. Peatland in old lake basin	Pohlia papillosa	3/13/10	5359
CHI4	CHI4_2	Cundinamarca, Fomeque, PNN Chingaza, Peatland on margins of Laguna de Chingaza. 4.5189 N, −73.7493 W, 3185 m.a.s.l.	Lake fen. Peatland in old lake basin	Noteroclada confluens	3/13/10	5360
CHI4	CHI4_6	Cundinamarca, Fomeque, PNN Chingaza, Peatland on margins of Laguna de Chingaza. 4.5189 N, −73.7493 W, 3185 m.a.s.l.	Lake fen. Peatland in old lake basin	Fossombronia sp	3/13/10	5361
CHI4	CHI4_12	Cundinamarca, Fomeque, PNN Chingaza, Peatland on margins of Laguna de Chingaza. 4.5189 N, −73.7493 W, 3185 m.a.s.l.	Lake fen. Peatland in old lake basin	Marchantia berteroana	3/13/10	5362
CHI5	CHI5_7	Cundinamarca, Fomeque, PNN Chingaza, Peatland on Espeletia Valley "Valle De Los Frailejones". 4.528 N, −73.7683 W, 3190 m.a.s.l.	Slope fen. Peatland in large valley in gentle slope	Riccardia paramorum	3/13/10	5363
CHI5	CHI5_8	Cundinamarca, Fomeque, PNN Chingaza, Peatland on Espeletia Valley "Valle De Los Frailejones". 4.528 N, −73.7683 W, 3190 m.a.s.l.	Slope fen. Peatland in large valley in gentle slope	Cephalozia sp	3/13/10	5364
CHI6	CHI6_5	Cundinamarca, Fomeque, PNN Chingaza, Peatland on Espeletia Valley "Valle De Los Frailejones". 4.5319 N, −73.7644 W, 3183 m.a.s.l.	Slope fen. Peatland in large valley in gentle slope	Lophocolea sp	3/13/10	5365

CHI7	CHI7_13	Cundinamarca, Fomeque, PNN Chingaza, Peatland on Espeletia Valley "Valle De Los Frailejones". 4.5358 N, −73.7636 W, 3182 m.a.s.l.	Slope fen. Peatland in large valley in gentle slope mix with very fine sediments	Sphagnum oxyphyllum	3/13/10	5366
CHI8	CHI8_10	Cundinamarca, Fomeque, PNN Chingaza, Peatland on Espeletia Valley "Valle De Los Frailejones". 4.5308 N, −73.7693 W, 3188 m.a.s.l.	Slope fen. Peatland in large valley in gentle slope	Riccardia paramorum	3/14/10	5367
CHI8	CHI8_18	Cundinamarca, Fomeque, PNN Chingaza, Peatland on Espeletia Valley "Valle De Los Frailejones". 4.5308 N, −73.7693 W, 3188 m.a.s.l.	Slope fen. Peatland in large valley in gentle slope	Cephalozia sp	3/14/10	5368
CHI9	CHI9_4	Cundinamarca, Fomeque, PNN Chingaza, Peatland on west side of "Valle De Los Frailejones". 4.5299 N, −73.7678 W, 3184 m.a.s.l.	Slope fen. Peatland in large valley in gentle slope	Sphagnum magellanicum	3/14/10	5369
CHI9	CHI9_5	Cundinamarca, Fomeque, PNN Chingaza, Peatland on west side of "Valle De Los Frailejones". 4.5299 N, −73.7678 W, 3184 m.a.s.l.	Slope fen. Peatland in large valley in gentle slope	Polytrichum commune	3/14/10	5370
CHI9	CHI9_9	Cundinamarca, Fomeque, PNN Chingaza, Peatland on west side of "Valle De Los Frailejones". 4.5299 N, −73.7678 W, 3184 m.a.s.l.	Slope fen. Peatland in large valley in gentle slope	Telaranea nematodes	3/14/10	5371
COC2	COC2_5	Boyaca, Guican. Vallle de los cojines. 6.4786 N, −72.2785 W, 4202 m.a.s.l.	Stream fen. Large fen with cushion plants	Marsupella sp	1/6/10	5372
COC2	COC2_5	Boyaca, Guican. Vallle de los cojines. 6.4786 N, −72.2785 W, 4202 m.a.s.l.	Stream fen. Large fen with cushion plants	Trichostomum brachydontium	1/6/10	5372

COC8	COC8_19	Boyaca, Guican. Vallle de los cojines. 6.4699 N, −72.2776 W, 4230 m.a.s.l.	Stream fen. Large fen with cushion plants in between the two streams on the south side of the complex	Hymenostylium recurvirostrum	1/8/10	5373
COC9	COC9_23	Boyaca, Guican. Vallle de los cojines. 6.4763 N, −72.2792 W, 4214 m.a.s.l.	Stream fen. Fen on floodplain of stream dominated by grasses and sedges with mineral soil mixed	Leptodontium luteum	1/8/10	5374
COC9	COC9_24	Boyaca, Guican. Vallle de los cojines. 6.4763 N, −72.2792 W, 4214 m.a.s.l.	Stream fen. Fen on floodplain of stream dominated by grasses and sedges with mineral soil mixed	Thuidium peruvianum	1/8/10	5375
COC9	COC9_26	Boyaca, Guican. Vallle de los cojines. 6.4763 N, −72.2792 W, 4214 m.a.s.l.	Stream fen. Fen on floodplain of stream dominated by grasses and sedges with mineral soil mixed	Pohlia papillosa	1/8/10	5376
MA1	MA1_3	Mamapacha, lake "La Tarea". 5.1918 N, −73.2649 W, 3059 m.a.s.l.	Slope fen. Lake fen, 50 m W from lake. Lake water level was raised around 8 m 10 years ago with a dam.	Riccardia paramorum	2/17/09	3852
MA1	MA1_3	Mamapacha, lake "La Tarea". 5.1918 N, −73.2649 W, 3059 m.a.s.l.	Slope fen. Lake fen, 50 m W from lake. Lake water level was raised around 8 m 10 years ago with a dam.	Riccardia paramorum	2/17/09	3878
MA1	MA1_4	Mamapacha, lake "La Tarea". 5.1918 N, −73.2649 W, 3059 m.a.s.l.	Slope fen. Lake fen, 50 m W from lake. Lake water level was raised around 8 m 10 years ago with a dam.	Sphagnum sp	2/17/09	3853
MA1	MA1_4	Mamapacha, lake "La Tarea". 5.1918 N, −73.2649 W, 3059 m.a.s.l.	Slope fen. Lake fen, 50 m W from lake. Lake water level was raised around 8 m 10 years ago with a dam.	Sphagnum sp	2/17/09	3879
MA1	MA1_5	Mamapacha, lake "La Tarea". 5.1918 N, −73.2649 W, 3059 m.a.s.l.	Slope fen. Lake fen, 50 m W from lake. Lake water level was raised around 8 m 10 years ago with a dam.	Thuidium peruvianum	2/17/09	3854

MA1	MA1_5	Mamapacha, lake "La Tarea". 5.1918 N, −73.2649 W, 3059 m.a.s.l.	Slope fen. Lake fen, 50 m W from lake. Lake water level was raised around 8 m 10 years ago with a dam.	Thuidium peruvianum	2/17/09	3880
MA1	MA1_6	Mamapacha, lake "La Tarea". 5.1918 N, −73.2649 W, 3059 m.a.s.l.	Slope fen. Lake fen, 50 m W from lake. Lake water level was raised around 8 m 10 years ago with a dam.	Frullania sp	2/17/09	3881
MA1	MA1_6	Mamapacha, lake "La Tarea". 5.1918 N, −73.2649 W, 3059 m.a.s.l.	Slope fen. Lake fen, 50 m W from lake. Lake water level was raised around 8 m 10 years ago with a dam.	Frullania sp	2/17/09	3855
MA1	MA1_7	Mamapacha, lake "La Tarea". 5.1918 N, −73.2649 W, 3059 m.a.s.l.	Slope fen. Lake fen, 50 m W from lake. Lake water level was raised around 8 m 10 years ago with a dam.	Breutelia chrysea	2/17/09	3856
MA1	MA1_7	Mamapacha, lake "La Tarea". 5.1918 N, −73.2649 W, 3059 m.a.s.l.	Slope fen. Lake fen, 50 m W from lake. Lake water level was raised around 8 m 10 years ago with a dam.	Breutelia chrysea	2/17/09	3882
MA1	MA1_10	Mamapacha, lake "La Tarea". 5.1918 N, −73.2649 W, 3059 m.a.s.l.	Slope fen. Lake fen, 50 m W from lake. Lake water level was raised around 8 m 10 years ago with a dam.	Bartramia sp	2/17/09	3859
MA1	MA1_10	Mamapacha, lake "La Tarea". 5.1918 N, −73.2649 W, 3059 m.a.s.l.	Slope fen. Lake fen, 50 m W from lake. Lake water level was raised around 8 m 10 years ago with a dam.	Bartramia sp	2/17/09	3885
MA10	MA10_7	Mamapacha, lake "Los Patos". 5.1876 N, −73.272 W, 3145 m.a.s.l.	Stream fen. Stream fen on spill of Lake los patos	Brachytecium austroglareosum	2/22/09	3929
MA10	MA10_8	Mamapacha, lake "Los Patos". 5.1876 N, −73.272 W, 3145 m.a.s.l.	Stream fen. Stream fen on spill of Lake los patos	Aneura sp	2/22/09	3930
MA10	MA10_10	Mamapacha, lake "Los Patos". 5.1876 N, −73.272 W, 3145 m.a.s.l.	Stream fen. Stream fen on spill of Lake los patos	Marchantia berteroana	2/22/09	3932

MA11	MA11_21	Mamapacha, 300 m from way to Lake Lajadilla. 5.1864 N, −73.2701 W, 3180 m.a.s.l.	Slope fen. Small fen at outlet of small watershed less than 5 ha	Anastrophyllum sp	2/22/09	3934
MA2	MA2_8	Mamapacha, lake "La Tarea". 5.1918 N, −75.2652 W, 3061 m.a.s.l.	Slope fen. Slope fen similar to MA1 with sphagnum in the hollows and chusquea forming hummocks	Moss	2/17/09	3887
MA3	MA3_4	Mamapacha, lake "La Tarea". 5.19 N, −73.2639 W, 3064 m.a.s.l.	Lake fen. Lake fen, floating mat on lake anchored to rock outcrop	Cephalozia sp	2/18/09	3888
MA3	MA3_7	Mamapacha, lake "La Tarea". 5.19 N, −73.2639 W, 3064 m.a.s.l.	Lake fen. Lake fen, floating mat on lake anchored to rock outcrop	Herbertus divergens	2/18/09	3889
MA3	MA3_11	Mamapacha, lake "La Tarea". 5.19 N, −73.2639 W, 3064 m.a.s.l.	Lake fen. Lake fen, floating mat on lake anchored to rock outcrop	Isotachis sp	2/18/09	3890
MA3	MA3_15	Mamapacha, lake "La Tarea". 5.19 N, −73.2639 W, 3064 m.a.s.l.	Lake fen. Lake fen, floating mat on lake anchored to rock outcrop	Campylopus arctocarpus	2/18/09	3891
MA4	MA4_5	Mamapacha, lake "La Tarea". 5.1895 N, −73.2639 W, 3064 m.a.s.l.	Lake fen. Lake fen, floating mat on lake anchored to rock outcrop	Drepanocladus aduncus	2/18/09	3892
MA4	MA4_12	Mamapacha, lake "La Tarea". 5.1895 N, −73.2639 W, 3064 m.a.s.l.	Lake fen. Lake fen, floating mat on lake anchored to rock outcrop	Plachiochila sp	2/18/09	3893
MA5	MA5_2	Mamapacha, lake "Los Patos". 5.1899 N, −73.2736 W, 3154 m.a.s.l.	Lake fen. Fen with chusquea similar to a forested fen but with chusquea	Sphagnum sp	2/19/09	3894
MA5	MA5_6	Mamapacha, lake "Los Patos". 5.1899 N, −73.2736 W, 3154 m.a.s.l.	Lake fen. Fen with chusquea similar to a forested fen but with chusquea	Dicranella sp	2/19/09	3895
MA5	MA5_7	Mamapacha, lake "Los Patos". 5.1899 N, −73.2736 W, 3154 m.a.s.l.	Lake fen. Fen with chusquea similar to a forested fen but with chusquea	Campylopus arctocarpus	2/19/09	3896
MA6	MA6_6	Mamapacha, lake "Los Patos". 5.1902 N, −73.2743 W, 3155 m.a.s.l.	Lake fen. Fen with chusquea similar to a forested fen but with chusquea, drier than MA5. Just 50 m from MA5	Breutelia chrysea	2/19/09	4052

MA6	MA6_7	Mamapacha, lake "Los Patos". 5.1902 N, −73.2743 W, 3155 m.a.s.l.	Lake fen. Fen with chusquea similar to a forested fen but with chusquea, drier than MA5. Just 50 m from MA5	Aneura sp	2/19/09	3897
MA6	MA6_8	Mamapacha, lake "Los Patos". 5.1902 N, −73.2743 W, 3155 m.a.s.l.	Lake fen. Fen with chusquea similar to a forested fen but with chusquea, drier than MA5. Just 50 m from MA5	Adelanthus pittieri	2/19/09	3898
MA6	MA6_9	Mamapacha, lake "Los Patos". 5.1902 N, −73.2743 W, 3155 m.a.s.l.	Lake fen. Fen with chusquea similar to a forested fen but with chusquea, drier than MA5. Just 50 m from MA5	Odontoschisma sp	2/19/09	3899
MA6	MA6_14	Mamapacha, lake "Los Patos". 5.1902 N, −73.2743 W, 3155 m.a.s.l.	Lake fen. Fen with chusquea similar to a forested fen but with chusquea, drier than MA5. Just 50 m from MA5	Anastrophyllum nigrescens	2/19/09	4062
MA6	MA6_14	Mamapacha, lake "Los Patos". 5.1902 N, −73.2743 W, 3155 m.a.s.l.	Lake fen. Fen with chusquea similar to a forested fen but with chusquea, drier than MA5. Just 50 m from MA5	Anastrophyllum nigrescens	2/19/09	3900
MA6	MA6_18	Mamapacha, lake "Los Patos". 5.1902 N, −73.2743 W, 3155 m.a.s.l.	Lake fen. Fen with chusquea similar to a forested fen but with chusquea, drier than MA5. Just 50 m from MA5	Lepidozia sp	2/19/09	3901
MA6	MA6_21	Mamapacha, lake "Los Patos". 5.1902 N, −73.2743 W, 3155 m.a.s.l.	Lake fen. Fen with chusquea similar to a forested fen but with chusquea, drier than MA5. Just 50 m from MA5	Lepidozia sp	2/19/09	3902
MA6	MA6_25	Mamapacha, lake "Los Patos". 5.1902 N, −73.2743 W, 3155 m.a.s.l.	Lake fen. Fen with chusquea similar to a forested fen but with chusquea, drier than MA5. Just 50 m from MA5	Riccardia paramorum	2/19/09	3905
MA7	MA7_1	Mamapacha. 5.197 N, −73.2732 W, 3154 m.a.s.l.	Lake fen. Fen dominated by sedges, with very little sphagnum. Small stream running through it and wide hummocks of chusquea over 2 m in diameter	Campylopus arctocarpus	2/20/09	3908

MA7	MA7_2	Mamapacha. 5.197 N, −73.2732 W, 3154 m.a.s.l.	Lake fen. Fen dominated by sedges, with very little sphagnum. Small stream running through it and wide hummocks of chusquea over 2 m in diameter	Breutelia chrysea	2/20/09	3909
MA7	MA7_7	Mamapacha. 5.197 N, −73.2732 W, 3154 m.a.s.l.	Lake fen. Fen dominated by sedges, with very little sphagnum. Small stream running through it and wide hummocks of chusquea over 2 m in diameter	Herbertus divergens	2/20/09	3910
MA7	MA7_9	Mamapacha. 5.197 N, −73.2732 W, 3154 m.a.s.l.	Lake fen. Fen dominated by sedges, with very little sphagnum. Small stream running through it and wide hummocks of chusquea over 2 m in diameter	Riccardia paramorum	2/20/09	3912
MA7	MA7_13	Mamapacha. 5.197 N, −73.2732 W, 3154 m.a.s.l.	Lake fen. Fen dominated by sedges, with very little sphagnum. Small stream running through it and wide hummocks of chusquea over 2 m in diameter	Dicranella sp	2/20/09	3914
MA7	MA7_21	Mamapacha. 5.197 N, −73.2732 W, 3154 m.a.s.l.	Lake fen. Fen dominated by sedges, with very little sphagnum. Small stream running through it and wide hummocks of chusquea over 2 m in diameter	Nardia succulenta	2/20/09	3919
MA7	MA7_23	Mamapacha. 5.197 N, −73.2732 W, 3154 m.a.s.l.	Lake fen. Fen dominated by sedges, with very little sphagnum. Small stream running through it and wide hummocks of chusquea over 2 m in diameter	Bryum laevigatum	2/20/09	3920
MA8	MA8_10	Mamapacha, Lake "Lajadilla". 5.1842 N, −73.2749 W, 3240 m.a.s.l.	Stream fen. Small fen near to Lajadilla lake, fen formed by ridge stopping the flow of water towards the lake	Plachiochila sp	2/21/09	3922

MA8	MA8_13	Mamapacha, Lake "Lajadilla". 5.1842 N, −73.2749 W, 3240 m.a.s.l.	Stream fen. Small fen near to Lajadilla lake, fen formed by ridge stopping the flow of water towards the lake	Bazzania sp	2/21/09	3924
MA8	MA8_14	Mamapacha, Lake "Lajadilla". 5.1842 N, −73.2749 W, 3240 m.a.s.l.	Stream fen. Small fen near to Lajadilla lake, fen formed by ridge stopping the flow of water towards the lake	Anastrophyllum nigrescens	2/21/09	3925
MA9	MA9_18	Mamapacha, Lake "Lajadilla". 5.1928 N, −73.2724 W, 3238 m.a.s.l.	Stream fen. Small fen near to Lajadilla lake, fen formed by ridge stopping the flow of water towards the lake	Moss	2/21/09	3928
SUM1	SUM1_2	Sumapaz. Road to Nazareth. Fen close to trout fishery at roadside. 4.2594 N, −74.1882 W, 3526 m.a.s.l.	Slope fen. Slope fen with a small stream (< 10 cm wide) running through	Breutelia chrysea	1/27/09	3935
SUM1	SUM1_2	Sumapaz. Road to Nazareth. Fen close to trout fishery at roadside. 4.2594 N, −74.1882 W, 3526 m.a.s.l.	Slope fen. Slope fen with a small stream (< 10 cm wide) running through	Breutelia chrysea	1/27/09	4056
SUM1	SUM1_3	Sumapaz. Road to Nazareth. Fen close to trout fishery at roadside. 4.2594 N, −74.1882 W, 3526 m.a.s.l.	Slope fen. Slope fen with a small stream (< 10 cm wide) running through	Campylopus andersonii	1/27/09	3936
SUM1	SUM1_3	Sumapaz. Road to Nazareth. Fen close to trout fishery at roadside. 4.2594 N, −74.1882 W, 3526 m.a.s.l.	Slope fen. Slope fen with a small stream (< 10 cm wide) running through	Campylopus andersonii	1/27/09	4057
SUM1	SUM1_8	Sumapaz. Road to Nazareth. Fen close to trout fishery at roadside. 4.2594 N, −74.1882 W, 3526 m.a.s.l.	Slope fen. Slope fen with a small stream (< 10 cm wide) running through	Marchantia berteroana	1/27/09	3940
SUM1	SUM1_11	Sumapaz. Road to Nazareth. Fen close to trout fishery at roadside. 4.2594 N, −74.1882 W, 3526 m.a.s.l.	Slope fen. Slope fen with a small stream (< 10 cm wide) running through	Hypnum cupressiforme	1/27/09	3943

SUM1	SUM1_13	Sumapaz. Road to Nazareth. Fen close to trout fishery at roadside. 4.2594 N, −74.1882 W, 3526 m.a.s.l.	Slope fen. Slope fen with a small stream (< 10 cm wide) running through	Warnstorfia exannulata	1/27/09	3945
SUM11	SUM11_4	Sumapaz. Road to Nazareth. Fen close to trout fishery at roadside. 4.2759 N, −74.1969 W, 3686 m.a.s.l.	Stream fen. Sphagnum blankets in river floodplain extending until valley margins	Polytrichum commune	2/1/09	4010
SUM13	SUM13_8	Sumapaz. Road to Nazareth. Fen close to trout fishery at roadside. 4.3172 N, −74.2056 W, 3580 m.a.s.l.	Slope fen.	Thuidium peruvianum	2/2/09	4014
SUM13	SUM13_17	Sumapaz. Road to Nazareth. Fen close to trout fishery at roadside. 4.3172 N, −74.2056 W, 3580 m.a.s.l.	Slope fen.	Nardia succulenta	2/2/09	4015
SUM14	SUM14_18	Sumapaz. Road to Nazareth. Fen close to trout fishery at roadside. 4.3172 N, −74.2056 W, 3530 m.a.s.l.	Slope fen.	Brachytecium austroglareosum	2/3/09	4019
SUM2	SUM2_1	Sumapaz. Road to Nazareth. Fen close to trout fishery at roadside. 4.2613 N, −74.1909 W, 3552 m.a.s.l.	Slope fen. Slope fen with a small stream (< 10 cm wide) running through	Campylopus arctocarpus	1/27/09	3946
SUM2	SUM2_3	Sumapaz. Road to Nazareth. Fen close to trout fishery at roadside. 4.2613 N, −74.1909 W, 3552 m.a.s.l.	Slope fen. Slope fen with a small stream (< 10 cm wide) running through	Sphagnum sancto-josephense	1/27/09	3947
SUM2	SUM2_8	Sumapaz. Road to Nazareth. Fen close to trout fishery at roadside. 4.2613 N, −74.1909 W, 3552 m.a.s.l.	Slope fen. Slope fen with a small stream (< 10 cm wide) running through	Drepanocladus aduncus	1/27/09	3949
SUM2	SUM2_10	Sumapaz. Road to Nazareth. Fen close to trout fishery at roadside. 4.2613 N, −74.1909 W, 3552 m.a.s.l.	Slope fen. Slope fen with a small stream (< 10 cm wide) running through	Lophocolea sp2	1/27/09	3950

SUM2	SUM2_12	Sumapaz. Road to Nazareth. Fen close to trout fishery at roadside. 4.2613 N, −74.1909 W, 3552 m.a.s.l.	Slope fen. Slope fen with a small stream (< 10 cm wide) running through	Sphagnum magellanicum	1/27/09	3952
SUM2	SUM2_13	Sumapaz. Road to Nazareth. Fen close to trout fishery at roadside. 4.2613 N, −74.1909 W, 3552 m.a.s.l.	Slope fen. Slope fen with a small stream (< 10 cm wide) running through	Sphagnum cundinamarcanum	1/27/09	3953
SUM2	SUM2_15	Sumapaz. Road to Nazareth. Fen close to trout fishery at roadside. 4.2613 N, −74.1909 W, 3552 m.a.s.l.	Slope fen. Slope fen with a small stream (< 10 cm wide) running through	Riccardia paramorum	1/27/09	3954
SUM3	SUM3_3	Sumapaz. Road to Nazareth. Fen close to trout fishery at roadside. 4.2759 N, −74.1969 W, 3569 m.a.s.l.	Slope fen.	Breutelia chrysea	1/28/09	3955
SUM3	SUM3_5	Sumapaz. Road to Nazareth. Fen close to trout fishery at roadside. 4.2759 N, −74.1969 W, 3569 m.a.s.l.	Slope fen.	Sphagnum rio-negrense	1/28/09	3957
SUM3	SUM3_11	Sumapaz. Road to Nazareth. Fen close to trout fishery at roadside. 4.2759 N, −74.1969 W, 3569 m.a.s.l.	Slope fen.	Cephalozia sp	1/28/09	3959
SUM3	SUM3_12	Sumapaz. Road to Nazareth. Fen close to trout fishery at roadside. 4.2759 N, −74.1969 W, 3569 m.a.s.l.	Slope fen.	Calliergon stramineum	1/28/09	3960
SUM3	SUM3_13	Sumapaz. Road to Nazareth. Fen close to trout fishery at roadside. 4.2759 N, −74.1969 W, 3569 m.a.s.l.	Slope fen.	Stephaniella paraphyllina	1/28/09	3961
SUM3	SUM3_14	Sumapaz. Road to Nazareth. Fen close to trout fishery at roadside. 4.2759 N, −74.1969 W, 3569 m.a.s.l.	Slope fen.	Cephalozia sp	1/28/09	3962

SUM3	SUM3_19	Sumapaz. Road to Nazareth. Fen close to trout fishery at roadside. 4.2759 N, −74.1969 W, 3569 m.a.s.l.	Slope fen.	Moss	1/28/09	3964
SUM3	SUM3_20	Sumapaz. Road to Nazareth. Fen close to trout fishery at roadside. 4.2759 N, −74.1969 W, 3569 m.a.s.l.	Slope fen.	Campylopus andersonii	1/28/09	3965
SUM3	SUM3_21	Sumapaz. Road to Nazareth. Fen close to trout fishery at roadside. 4.2759 N, −74.1969 W, 3569 m.a.s.l.	Slope fen.	Bryum laevigatum	1/28/09	3966
SUM4	SUM4_2	Sumapaz. Road to Nazareth. Fen close to trout fishery at roadside. 4.2759 N, −74.1969 W, 3667 m.a.s.l.	Slope fen.	Campylopus arctocarpus	1/28/09	3967
SUM4	SUM4_5	Sumapaz. Road to Nazareth. Fen close to trout fishery at roadside. 4.2759 N, −74.1969 W, 3667 m.a.s.l.	Slope fen.	Riccardia paramorum	1/28/09	3969
SUM4	SUM4_6	Sumapaz. Road to Nazareth. Fen close to trout fishery at roadside. 4.2759 N, −74.1969 W, 3667 m.a.s.l.	Slope fen.	Sphagnum compactum	1/28/09	3970
SUM4	SUM4_9	Sumapaz. Road to Nazareth. Fen close to trout fishery at roadside. 4.2759 N, −74.1969 W, 3667 m.a.s.l.	Slope fen.	Anastrophyllum nigrescens	1/28/09	3971
SUM4	SUM4_11	Sumapaz. Road to Nazareth. Fen close to trout fishery at roadside. 4.2759 N, −74.1969 W, 3667 m.a.s.l.	Slope fen.	Jamesoniella rubricaulis	1/28/09	3972
SUM4	SUM4_14	Sumapaz. Road to Nazareth. Fen close to trout fishery at roadside. 4.2759 N, −74.1969 W, 3667 m.a.s.l.	Slope fen.	Lycopodiella glaucescens	1/28/09	3973

SUM5	SUM5_1	Sumapaz. Road to Nazareth. Fen close to trout fishery at roadside. 4.2759 N, −74.1969 W, 3415 m.a.s.l.	Slope fen.	Sphagnum sancto-josephense	1/29/09	3974
SUM5	SUM5_10	Sumapaz. Road to Nazareth. Fen close to trout fishery at roadside. 4.2759 N, −74.1969 W, 3415 m.a.s.l.	Slope fen.	Cephalozia sp	1/29/09	3977
SUM5	SUM5_12	Sumapaz. Road to Nazareth. Fen close to trout fishery at roadside. 4.2759 N, −74.1969 W, 3415 m.a.s.l.	Slope fen.	Moss	1/29/09	3978
SUM6	SUM6_6	Sumapaz. Road to Nazareth. Fen close to trout fishery at roadside. 4.2759 N, −74.1969 W, 3701 m.a.s.l.	Slope fen. Highly disturbed slope fen, in outflow from Lake "Laguna Negra"	Cephalozia sp	1/29/09	3979
SUM6	SUM6_7	Sumapaz. Road to Nazareth. Fen close to trout fishery at roadside. 4.2759 N, −74.1969 W, 3701 m.a.s.l.	Slope fen. Highly disturbed slope fen, in outflow from Lake "Laguna Negra"	Moss	1/29/09	3980
SUM6	SUM6_8	Sumapaz. Road to Nazareth. Fen close to trout fishery at roadside. 4.2759 N, −74.1969 W, 3701 m.a.s.l.	Slope fen. Highly disturbed slope fen, in outflow from Lake "Laguna Negra"	Polytrichum commune	1/29/09	3981
SUM6	SUM6_11	Sumapaz. Road to Nazareth. Fen close to trout fishery at roadside. 4.2759 N, −74.1969 W, 3701 m.a.s.l.	Slope fen. Highly disturbed slope fen, in outflow from Lake "Laguna Negra"	Polytrichum commune	1/29/09	3982
SUM6	SUM6_20	Sumapaz. Road to Nazareth. Fen close to trout fishery at roadside. 4.2759 N, −74.1969 W, 3701 m.a.s.l.	Slope fen. Highly disturbed slope fen, in outflow from Lake "Laguna Negra"	Fossombronia sp	1/29/09	3986
SUM6	SUM6_21	Sumapaz. Road to Nazareth. Fen close to trout fishery at roadside. 4.2759 N, −74.1969 W, 3701 m.a.s.l.	Slope fen. Highly disturbed slope fen, in outflow from Lake "Laguna Negra"	Ditrichum sp	1/29/09	3987

SUM6	SUM6_23	Sumapaz. Road to Nazareth. Fen close to trout fishery at roadside. 4.2759 N, −74.1969 W, 3701 m.a.s.l.	Slope fen. Highly disturbed slope fen, in outflow from Lake "Laguna Negra"	Sphagnum oxyphyllum	1/29/09	3988
SUM6	SUM6_25	Sumapaz. Road to Nazareth. Fen close to trout fishery at roadside. 4.2759 N, −74.1969 W, 3701 m.a.s.l.	Slope fen. Highly disturbed slope fen, in outflow from Lake "Laguna Negra"	Sphagnum cyclophyllum	1/29/09	3989
SUM6	SUM6_26	Sumapaz. Road to Nazareth. Fen close to trout fishery at roadside. 4.2759 N, −74.1969 W, 3701 m.a.s.l.	Slope fen. Highly disturbed slope fen, in outflow from Lake "Laguna Negra"	Stephaniella paraphyllina	1/29/09	3990
SUM6	SUM6_28	Sumapaz. Road to Nazareth. Fen close to trout fishery at roadside. 4.2759 N, −74.1969 W, 3701 m.a.s.l.	Slope fen. Highly disturbed slope fen, in outflow from Lake "Laguna Negra"	Bryum laevigatum	1/29/09	3991
SUM7	SUM7_3	Sumapaz. Road to Nazareth. Fen close to trout fishery at roadside. 4.2759 N, −74.1969 W, 3645 m.a.s.l.	Lake fen. Small glacier cirques with small lake, peatland developed from terrestrialization of the lake	Brachytecium sp	1/30/09	3993
SUM8	SUM8_6	Sumapaz. Road to Nazareth. Fen close to trout fishery at roadside. 4.2759 N, −74.1969 W, 3616 m.a.s.l.	Stream fen. Fen developed from small stream running from lake "Laguna Negra"	Sphagnum magellanicum	1/30/09	3995
SUM8	SUM8_11	Sumapaz. Road to Nazareth. Fen close to trout fishery at roadside. 4.2759 N, −74.1969 W, 3616 m.a.s.l.	Stream fen. Fen developed from small stream running from lake "Laguna Negra"	Campylopus arctocarpus	1/30/09	3996
SUM8	SUM8_15	Sumapaz. Road to Nazareth. Fen close to trout fishery at roadside. 4.2759 N, −74.1969 W, 3616 m.a.s.l.	Stream fen. Fen developed from small stream running from lake "Laguna Negra"	Dicranella sp	1/30/09	3999
SUM8	SUM8_16	Sumapaz. Road to Nazareth. Fen close to trout fishery at roadside. 4.2759 N, −74.1969 W, 3616 m.a.s.l.	Stream fen. Fen developed from small stream running from lake "Laguna Negra"	Campylopus sp	1/30/09	4000

SUM9	SUM9_11	Sumapaz. Road to Nazareth. Fen close to trout fishery at roadside. 4.2759 N, −74.1969 W, 3621 m.a.s.l.	Raised bog. Bog dome developed from small stream running from lake "Laguna Negra"	Isotachis sp	1/30/09	4002
SUM9	SUM9_16	Sumapaz. Road to Nazareth. Fen close to trout fishery at roadside. 4.2759 N, −74.1969 W, 3621 m.a.s.l.	Raised bog. Bog dome developed from small stream running from lake "Laguna Negra"	Lepidozia sp	1/30/09	4004
SUM9	SUM9_18	Sumapaz. Road to Nazareth. Fen close to trout fishery at roadside. 4.2759 N, −74.1969 W, 3621 m.a.s.l.	Raised bog. Bog dome developed from small stream running from lake "Laguna Negra"	Campylopus arctocarpus	1/30/09	4006
SUM9	SUM9_20	Sumapaz. Road to Nazareth. Fen close to trout fishery at roadside. 4.2759 N, −74.1969 W, 3621 m.a.s.l.	Raised bog. Bog dome developed from small stream running from lake "Laguna Negra"	Riccardia paramorum	1/30/09	4008
BE13	BE13_XX	Antioquia, Belmira, Paramo de Belmira, Sitio La Laguna. 6.6722 N, −75.6685 W, 3270 m.a.s.l.	Stream fen. Large raised peatland in upland plateau		6/26/10	5469
BE11	BE11_8	Antioquia, Belmira, Paramo de Belmira, Sitio La Laguna. 6.6668 N, −75.6691 W, 3269 m.a.s.l.	Stream fen. Large raised peatland in upland plateau	Isoetes sp	2/24/10	5470
BE11	BE11_X1	Antioquia, Belmira, Paramo de Belmira, Sitio La Laguna. 6.6668 N, −75.6691 W, 3269 m.a.s.l.	Stream fen. Large raised peatland in upland plateau		2/24/10	5471
BE11	BE11_WP34	Antioquia, Belmira, Paramo de Belmira, Sitio La Laguna. 6.6668 N, −75.6691 W, 3269 m.a.s.l.	Stream fen. Large raised peatland in upland plateau		2/24/10	5472
BE11	BE11_X2	Antioquia, Belmira, Paramo de Belmira, Sitio La Laguna. 6.6668 N, −75.6691 W, 3269 m.a.s.l.	Stream fen. Large raised peatland in upland plateau		2/24/10	5473

CHI1	CHI1_ JCB4650	Cundinamarca, Fomeque, PNN Chingaza, Heads of "Rio Frio", Laguna de Chingaza. 4.501 N, −73.7531 W, 3333 m.a.s.l.	Stream fen. Peatland in backwater floodplain from Rio Frio margins		3/12/10	4650
CHI1	CHI1_ JCB4651	Cundinamarca, Fomeque, PNN Chingaza, Heads of "Rio Frio", Laguna de Chingaza. 4.501 N, −73.7531 W, 3333 m.a.s.l.	Stream fen. Peatland in backwater floodplain from Rio Frio margins		3/12/10	4651
CHI1	CHI1_ JCB4652	Cundinamarca, Fomeque, PNN Chingaza, Heads of "Rio Frio", Laguna de Chingaza. 4.501 N, −73.7531 W, 3333 m.a.s.l.	Stream fen. Peatland in backwater floodplain from Rio Frio margins		3/12/10	4652
CHI1	CHI1_ JCB4653	Cundinamarca, Fomeque, PNN Chingaza, Heads of "Rio Frio", Laguna de Chingaza. 4.501 N, −73.7531 W, 3333 m.a.s.l.	Stream fen. Peatland in backwater floodplain from Rio Frio margins		3/12/10	4653
CHI1	CHI1_ JCB4654	Cundinamarca, Fomeque, PNN Chingaza, Heads of "Rio Frio", Laguna de Chingaza. 4.501 N, −73.7531 W, 3333 m.a.s.l.	Stream fen. Peatland in backwater floodplain from Rio Frio margins		3/12/10	4654
CHI1	CHI1_ JCB4655	Cundinamarca, Fomeque, PNN Chingaza, Heads of "Rio Frio", Laguna de Chingaza. 4.501 N, −73.7531 W, 3333 m.a.s.l.	Stream fen. Peatland in backwater floodplain from Rio Frio margins		3/12/10	4655
CHI1	CHI1_ JCB4656	Cundinamarca, Fomeque, PNN Chingaza, Heads of "Rio Frio", Laguna de Chingaza. 4.501 N, −73.7531 W, 3333 m.a.s.l.	Stream fen. Peatland in backwater floodplain from Rio Frio margins		3/12/10	4656

COC11	COC11_ JCB4701	Boyaca, Guican. Vallle de los cojines. 6.4802 N, −72.2806 W, 4217 m.a.s.l.	Stream fen. Large fen with cushion plants in between the two streams on the south side of the complex		1/8/10	4701
CHI1	CHI1_X1	Cundinamarca, Fomeque, PNN Chingaza, Heads of “Rio Frio”, Laguna de Chingaza. 4.501 N, −73.7531 W, 3333 m.a.s.l.	Stream fen. Peatland in backwater floodplain from Rio Frio margins		3/12/10	4702
CHI1	CHI1_X2	Cundinamarca, Fomeque, PNN Chingaza, Heads of “Rio Frio”, Laguna de Chingaza. 4.501 N, −73.7531 W, 3333 m.a.s.l.	Stream fen. Peatland in backwater floodplain from Rio Frio margins		3/12/10	4703
COC11	COC11_X1	Boyaca, Guican. Vallle de los cojines. 6.4802 N, −72.2806 W, 4217 m.a.s.l.	Stream fen. Large fen with cushion plants in between the two streams on the south side of the complex		1/8/10	4704
COC11	COC11_X2	Boyaca, Guican. Vallle de los cojines. 6.4802 N, −72.2806 W, 4217 m.a.s.l.	Stream fen. Large fen with cushion plants in between the two streams on the south side of the complex		1/8/10	4705
COC11	COC11_X3	Boyaca, Guican. Vallle de los cojines. 6.4802 N, −72.2806 W, 4217 m.a.s.l.	Stream fen. Large fen with cushion plants in between the two streams on the south side of the complex		1/8/10	4706
COC11	COC11_X4	Boyaca, Guican. Vallle de los cojines. 6.4802 N, −72.2806 W, 4217 m.a.s.l.	Stream fen. Large fen with cushion plants in between the two streams on the south side of the complex		1/8/10	4707
COC11	COC11_X5	Boyaca, Guican. Vallle de los cojines. 6.4802 N, −72.2806 W, 4217 m.a.s.l.	Stream fen. Large fen with cushion plants in between the two streams on the south side of the complex		1/8/10	4708
COC12	COC12_X6	Boyaca, Guican. Pantano de los Patos. 6.4948 N, −72.2882 W, 4493 m.a.s.l.	Plateau fen. Cushion plants in plateau fen		1/9/10	4709

COC12	COC12_X7	Boyaca, Guican. Pantano de los Patos. 6.4948 N, −72.2882 W, 4493 m.a.s.l.	Plateau fen. Cushion plants in plateau fen		1/9/10	4710
COC12	COC12_X8	Boyaca, Guican. Pantano de los Patos. 6.4948 N, −72.2882 W, 4493 m.a.s.l.	Plateau fen. Cushion plants in plateau fen		1/9/10	4711
COC12	COC12_X9	Boyaca, Guican. Pantano de los Patos. 6.4948 N, −72.2882 W, 4493 m.a.s.l.	Plateau fen. Cushion plants in plateau fen		1/9/10	4712
COC12	COC12_X10	Boyaca, Guican. Pantano de los Patos. 6.4948 N, −72.2882 W, 4493 m.a.s.l.	Plateau fen. Cushion plants in plateau fen		1/9/10	4713
COC1	COC1_X11	Boyaca, Guican. Vallle de los cojines. 6.4786 N, −72.2784 W, 4198 m.a.s.l.	Stream fen. Large fen with cushion plants		1/6/10	4714
COC12	COC12_X12	Boyaca, Guican. Pantano de los Patos. 6.4948 N, −72.2882 W, 4493 m.a.s.l.	Plateau fen. Cushion plants in plateau fen		1/9/10	4715
COC12	COC12_X13	Boyaca, Guican. Pantano de los Patos. 6.4948 N, −72.2882 W, 4493 m.a.s.l.	Plateau fen. Cushion plants in plateau fen		1/9/10	4716
COC11	COC11_X14	Boyaca, Guican. Vallle de los cojines. 6.4802 N, −72.2806 W, 4217 m.a.s.l.	Stream fen. Large fen with cushion plants in between the two streams on the south side of the complex		1/8/10	4717
COC11	COC11_X15	Boyaca, Guican. Vallle de los cojines. 6.4802 N, −72.2806 W, 4217 m.a.s.l.	Stream fen. Large fen with cushion plants in between the two streams on the south side of the complex		1/8/10	4718
COC11	COC11_X16	Boyaca, Guican. Vallle de los cojines. 6.4802 N, −72.2806 W, 4217 m.a.s.l.	Stream fen. Large fen with cushion plants in between the two streams on the south side of the complex		1/8/10	4719
COC11	COC11_X17	Boyaca, Guican. Vallle de los cojines. 6.4802 N, −72.2806 W, 4217 m.a.s.l.	Stream fen. Large fen with cushion plants in between the two streams on the south side of the complex		1/8/10	4720

COC11	COC11_X18	Boyaca, Guican. Vallle de los cojines. 6.4802 N, −72.2806 W, 4217 m.a.s.l.	Stream fen. Large fen with cushion plants in between the two streams on the south side of the complex		1/8/10	4721
COC11	COC11_X19	Boyaca, Guican. Vallle de los cojines. 6.4802 N, −72.2806 W, 4217 m.a.s.l.	Stream fen. Large fen with cushion plants in between the two streams on the south side of the complex		1/8/10	4722

Table S2. Fitted environmental vectors for all species, bryophytes only, and vascular plant species only on NMDS ordinations (Figure 3). r-square is goodness of fit of vector to environmental variable values. *p*-value is calculated as the proportion of values with larger r-square after 1000 permutations.

	Species	
**Variables**	**r-square**	***p*-value**
Conductivity	0.191	0.001
Elevation	**0.631**	**0.001**
pH	0.196	0.004

Table S3. PERMANOVA table for all vegetation types combined and for specific contrasts among all vegetation types differentiated for the vegetation of 121 Andean peatlands. Probabilities are corrected by the number of comparisons.

**Source**	**Df**	**SS**	**MSS**	**F.Model**	**R2**	**Pr(>F)**
Vegetation type	4	9.31	3.6454	11.207	0.26177	0.001
Residuals	117	32.42	0.2659			
Total	120	41.73	1			

Contrasts

Combination of vegetation groups	Sums Sqs	Mean Sqs	F Model	R2	*p* value corrected
Cushion–Grasses	3.12	3.12	11.96	0.23	1.00E-04
Cushion–Sedges	4.62	4.62	21.71	0.33	1.00E-04
Cushion–Sphagnum	4.56	4.56	16.46	0.26	1.00E-04
Cushion–True mosses	4.59	4.59	18.22	0.26	1.00E-04
Grasses–Sedges	1.42	1.42	5.8	0.13	1.00E-04
Grasses–Sphagnum	1.81	1.81	5.74	0.12	1.00E-04
Grasses–True mosses	1.82	1.82	6.43	0.12	1.00E-04
Sedges–Sphagnum	1.99	1.99	7.48	0.14	1.00E-04
Sedges–True mosses	1.65	1.65	6.89	0.12	1.00E-04
Sphagnum–True mosses	1.65	1.65	5.61	0.09	1.00E-04

Table S4. Mean percent cover of the 260 species found in four different peat forming vegetation types from 121 peatlands in the northern Andes of Colombia.

Species	Cushion	Sedges	Sphagnum	True mosses	Grasses
Adelanthus decipiens	0	0	0	3.5	0
Adelanthus pittieri	3	0	0	0	3
Agerati tinifolia	0	0	2*	1.5	0
Agrostis hanckea	0	0	0	0	1
Agrostis trichodes	0	0	0	6	0
Allobiella campanensis	0	0	2.5	0	0
Astrophyllum nigrescens	3.9*	0	4	2	1
Astrophyllum sp	0	0	0	5	0
Andrewsianthus sp	0	0	1	0	0
Aneura pinguis	2.7	3.6	0	4	0
Anthoceros sp	0	0	0	3	0
Anthoxanthum odoratum	1	0	0	0	0
Aongstroemia filiformis	0	3	1	0	0
Apiaceae sp	0	0	0	5	3
Apiaceae sp2	4	0	0	0	0
Apiaceae sp3	1	0	0	0	0
Aptychella proligera	0	0	0	1	0
Arachniopsis coactilis	3	1	1.3	0	0
Aragoa abieti	0	1.7	0	1	0
Arcitophyllum albidum	0	6.4	10	0	0
Arcytophyllum muticum	0	4.3	6	4.4*	0
Atractylocarpus longisetus	0	3	0	3	3
Azolla sp	0	0	0	6	0

Species	Cushion	Sedges	Sphagnum	True mosses	Grasses
Bacharis revoluta	0	1.7	0	0	0
Bacharis sp2	3	0	0	0	0
Bacharis tricuneata	0	0	2.7	1.5	0
Bartsia sp	2.8	1	1	1	1
Bazzania sp	0	3	0	1	0
Blechnum loxense	0	2	2.8*	1.3	0
Brachytecium austroglareosum	4	0	1	1	3
Breutelia chrysea	0	0	0	0*	0
Breutelia chrysea	6.9	5.3	4.2	6.1	4.8
Breutelia triae	0	1	0	0	0
Bryohumbertia filifolia	0	0	0	3	0
Bryum alpinum	3.5	0	0	0	0
Bryum argenteum	2.8	0	0	1	0
Bryum laevigatum	0	0	1	2	3
Calamagrostis effussa	2.1	5.4	6.1	3.7	6.4*
Calamagrostis fibrovagita	6	3.8	1	3.8	5
Calypogeia rhombifolia	0	0	2.5	4	0
Campylium polygamum	6	0	0	0	0
Campylophyllum halleri	2	0	0	0	3
Campylopus aerodyction	0	0	0	6	0
Campylopus cuspidatus	3.4	4.4*	4	4	2.9
Campylopus edithae	0	0	0	1	0
Campylopus nivalis	6.6	3.4	2.8	3.5	4.5
Campylopus pauper	0	1	8	3.2	0
Campylopus richardii	2.6	2	1	0	0

Species	Cushion	Sedges	Sphagnum	True mosses	Grasses
Campylopus sharpii	0	0	0	1	0
Campylopus sp	0	3	3	0	0
Campylopus tallulensis	0	0	3	9	0
Campylopus trichophylloides	0	0	0	2.5	0
Campylopus trivialis	4.8	0	2	4	0
Cardamine boriensis	0	2	0	2.8	0
Carex bonplandii	11	9.5	5	6.8	13.4
Carex pichinchensis	0	1	3	2	0
Carex pygmaea	4.3	5.4	2.8	1.7	11.5*
Carex sp	0	0	7	0	0
Carex sp2	7.3	0	0	0	0
Cariophyllum sp	8	0	0	0	0
Carophyllaceae indet	0	1	0	0	0
Castratella pilloseroides	0	0	2	0	0
Cavendishia bracteata	0	0	2	0	0
Cephalozia sp	0	0	2.3	1.7	1.3
Cephalozia sp2	1	0	0	0	0
Cerastium arvense	0	1	0	1.5	0
Cerastium cf subspicatum	0	0	0	4	0
Cerastium sp	3.3	0	0	3	0
Cerastium sp2	1.3	0	0	0	0
Chusquea tessellata	0	4	3.3	6.6	0
Clasmatocolea sp	1	0	0	0	0
Cocclidium sp	0	0	0	2	0
Commeli sp	0	0	2	0	0
Cortaderia nitida	1	4.1	5.3	6.6	2.2

Species	Cushion	Sedges	Sphagnum	True mosses	Grasses
Crassula venezulensis	3	1	0	2	0
Daltonia tenuifolia	1	0	0	0	0
Dicranum frigidum	0	1	0	1	0
Dicranum peruvianum	1	0	0	0	0
Didymodon rigidulus	0	0	0	1	0
Diplostephium phylicoides	2	0	0	0	0
Diplostephium revolutum	0	1.9	0	3.4	0
Diplostephium romeroi	0	0	3	0	3
Diplostephium sp	2	0	3	0	1
Diplostephium sp1	0	2	0	1	0
Diplostephium sp2	0	0	2.8	0	0
Disterigma alaternoides	0	1	0	3*	1
Distichia muscoides	18.8*	0	0	0	6
Ditrichum rufescens	0	0	0	1	3
Drepanocladus aduncus	2.5	2	1	2	5
Drepanocladus longifolius	0	0	0	1	0
Entodon sp	1	0	0	0	0
Equisetum bogotensis	0	0	0	1	1
Eriosorus sp	0	0	1.5	1	0
Erythrophyllastrum andinum	0	0	0	2	0
Escallonia myrtilloides	0	0	0	2.7	0
Espeletia argentea	0	1	0	0	0
Espeletia grandiflora	0	4.1*	3	1	2.5
Espeletia lopezii	1.3	0	0	0	0
Espeletia occidentalis	0	0	0	0	3.7*
Floscaldasia sp	2.5	0	0	0	0

Species	Cushion	Sedges	Sphagnum	True mosses	Grasses
Fossombronia peruvia	3.3	0	0	1	0
Fossombronia sp	0	0	0	4	0
Frullania sp	0	0	1	2	0
Galium ascendens	0	0	0	3.4	0
Galium sp	0	0	0	1	0
Gaultheria sp	0	0	3	0	0
Gentia sedifolia	2	1	2	0	1.4
Gentianella sp	2	0	2	0	0
Geranium multiceps	2	0	0	1	0
Geranium sibbaldioides	2.8	1	0	0	0
Gphalium paramonum	8	0	0	0	0
Gongylanthus gratensis	0	1	0	1	0
Gymnostomum aeruginosum	0	0	0	1	0
Gynoxis sp	0	1.3	0	0	0
Halenia gunerifolia	0	2.3	6	1	1
Herbertus divergens	0	0	3	1	0
Hesperomeles heterophylla	0	0	1	0	0
Hesperomeles sp	0	1	0	0	0
Huperzia crassa	1.2	3.3	0	2	1
Hydrocotile gunnerifolia	6	0	0	0	0
Hydrocotile sp	0	0	1.2	2.6	0
Hymenostylium recurvirostrum	3	0	0	0	0
Hypericum juniperinum	1.6	1.2	1	2	1.7
Hypericum mirtyfolium	0	1	0	0	0
Hypericum muticum	0	3	0	3.5	0

Species	Cushion	Sedges	Sphagnum	True mosses	Grasses
Hypericum myricaefolium	0	1	1	2.7	0
Hypericum niviefolium	0	0	0	0	2
Hypericum strictum	0	0	0	2.5	0
Hypericum vaccinioides	0	0	0	0	1
Hypnum cupressiforme	1	2	1.7	3.3*	0
Hypochaeris sessiliflora	4.3	0	0	0	4.6
Hypochaeris sp	1.2	0	2	0	1
Hypochaeris sp2	0	0	3	1	1
Hypochaeris sp3	0	0	0	0	1
Hypochaeris sp4	3.5	0	0	0	3.3
Hypochaeris sp5	0	0	0	0	4
Hypochaeris taraxacifolia	0	0	1.5	0	0
Isoetes sp	0	3	2	0	0
Isotachis multiceps	7.4	1.7	2	2.3	1
Jamesonia sp	0	0	0	1	1
Jamesoniella rubricaulis	0	4.2	3	2.7	5.3
Juncus breviculmis	0	3.3	5.5	1	0
Juncus effusus	0	0	1.9*	1	0
Jungermannia sp	3.8	0	0	0	0
Kurzia capillaris	0	1	2.5	2	1
Lachemilla mandonia	0	4	2	2.7	0
Lachemilla nivalis	1.8	0	1.8	4	3
lachemilla orbicularis	2	3.6	1	5	3
Lachemilla sp	3.7	0	1.5	1	0
Lachemilla sp2	4	0	0	0	0
Lachemilla sp3	0	0	0	0	1

Species	Cushion	Sedges	Sphagnum	True mosses	Grasses
Lepidozia macrocolea	0	2.8	0	3.2	2
Leptodontium flexifolium	1	0	0	1	0
Leptodontium luteum	0	0	0	2.5	0
Leptoscyphus porphyrius	0	1	0	1	0
Lobatiriccardia sp	1	0	0	0	1
Lophocolea sp	0	1	1	1	0
Lophocolea sp2	0	0	0	1	0
Lophozia cf anomala	0	0	0	1	0
Lophozia sp	3.5	1	4	0	1
Lophozia sp2	3*	0	0	0	1
Lophozia sp3	0	1	3.5	0	0
Loricaria complata	1	0	0	0	1
Lycopodiella glaucescens	0	0	2.1	3.7	1
Lysipomia sphagnophila	3	4.3	0	4.5	0
Marchantia berteroa	7	0	0	4	0
Marchantia breviloba	0	0	0	4	0
Marchantia polymorpha	0	0	0	3	0
Marsupella sp	1	0	0	0	5
Metzgeria sinuata	0	1	0	0	0
Miconia sp	0	1	0	0	0
Moni sp	0	1.5	0	0	0
Montia fonta	0	0	0	1	0
Muhlenbergia fastigiata	0	14	0	1.5	0
Myriophyllum aquaticum	3	0	0	2.3	0
rdia succulenta	2	0	0	3.5	0
Nertera gradensis	1	2.3	3.5*	2.8	3

Species	Cushion	Sedges	Sphagnum	True mosses	Grasses
Niphogeton terta	0	0	0	3	0
Noteroclada confluens	0	1	0	2.4	0
Odontoschisma sp	0	0	0	4	0
Omphalanthus filiformis	0	0	0	1	0
Oreobolus cleefi	0	0	0	2.6	3
Oreobolus sp	0	0	0	0	6.7*
Orthrosanthus chimboracensis	0	0	0	1	0
Paepalanthus colombianus	0	2.3	2.7*	2.3	2.8
Paepalanthus lodicoloides	0	0	5.3	2.5	0
Paspalum hirtum	0	0	1.8	0	0
Pentacalia vaccinioides	4	0	2.5	3	1
Peperomia sp	0	0	0	1	0
Pernettya prostrata	3	5.7*	3.6	2	6.4
Philonotis longiseta	0	1	1	0	5
Pilopogon longirostris	11	0	2	0	0
Plachiochila sp	0	0	1	3.5	0
Plantago australis	0	1	0	0	0
Plantago rigida	0	0	0	0	6.2
Pleurozium schreberi	3.6	8.7	1	6.2	8.5
Pohlia elongata	0	4	0	0	0
Pohlia papillosa	1	0	0	1	0
Polypodium sp	0	0	2	1	0
Polytrichum commune	2	4.7	2.3	3.8	0
Pseudocephalozia quadriloba	2.8	0	3	0	0
Pteridophyta indet	0	0	4	0	0

Species	Cushion	Sedges	Sphagnum	True mosses	Grasses
Pteris sp	0	0	1	0	0
Puya goudutia	0	2.5*	1.6	1.7	0
puya killipi	0	3	3	3	0
Puya roldanii	0	0	1.7	0	0
Puya santonii	0	1.8	2	2.4	0
Radula sp	1	0	0	0	3
Ranunculus flageliformis	8	3	0	4.5	3
Ranunculus sp	0	0	0	1	0
Rhacoccarpus purpurascens	4.5	4.5	0	4	8.1*
Rhodobryum longifolium	0	1	0	3	0
Rhynchospora oreoboloidea	1.9	3.3	0	4.4	6.6*
Ribes sp	1.5	0	0	3	6
Riccardia capillacea	0	0	0	3	0
Riccardia columbica	4	1	0	3	4.5
Riccardia papillata	8	0	0	0	0
Riccardia paramorum	5.7	5.9*	3.6	3.4	3.9
Riccardia regi	3.3	0	0	1	3
Riccia sp	0	0	1	0	0
Rubus sp	0	0	1	0	0
Rumex tolimensis	2	0	0	0	0
Selaginella sp	0	0	2	2	0
Sematophyllum swartzii	0	0	0	0	3
Senecio formosoides	1.2	0	0	0	0
Sisyrinchium sp	0	0	2	0	0
Solenostoma sphaerocarpoidea	0	0	0	1	0

Species	Cushion	Sedges	Sphagnum	True mosses	Grasses
Sphaerothecium phascoideum	0	0	4	0	0
Sphagnum angustifolium	0	0	11	9	6.5
Sphagnum boyacanum	0	0	0	2	0
Sphagnum caldense	0	0	0	6	0
Sphagnum capillifolium	0	0	9	3.5	0
Sphagnum cf antioquensis	0	0	2	0	0
Sphagnum cf falcatulum Besch.	0	10.3	15.5	13.5	25
Sphagnum cleefii	0	0	0	1	0
Sphagnum compactum	0	1	0	2.3	0
Sphagnum cundimarcanum	0	0	0	2.7	0
Sphagnum cyclophyllum	3	0	0	0	0
Sphagnum lescurii	0	4.6	2	1	0
Sphagnum magellanicum	0	9.4*	8.4	4.6	5.5
Sphagnum oxyphyllum	0	16.2	12	1	3.3
Sphagnum pylaesii	2	11	8	0	0
Sphagnum sancto-josephense	8	8.4	12.7	10.6*	9.8
Sphagnum sp nov	5.5	0	0	0	0
Sphagnum tenerum	0	3.7	0	0	10.3
Stachys cf eriantha	0	0	0	1	0
Stephaniella paraphylli	0	0	0	1	0
Straminergon stramineum	3	0	2.5	1.3	1
Symphyogy bogotensis	0	0	0	1	0
Symphyogy brasiliensis	1.7	0	3	0	1
Symphyogy brongniartii	0	0	2	0	0

Species	Cushion	Sedges	Sphagnum	True mosses	Grasses
Symphyogy podophylla	0	0	13	1	0
Sysirrinchium sp	0	0	0	3	0
Taraxacum officilis	0	0	1.5	0	0
Thuidium peruvianum	1	0	0	1.8	0
Trachyxiphium steerei	0	0	0	0	13
Trichostomum brachydontium	1.8	0	0	0	0
Utricularia sp	0	0	3	0	0
Valeria plantagiceae	2.3	1.9	0	1.3	3
Viola sp	3	0	0	0	0
Warnstorfia exannulata	4.6*	0	0	1	3
Werneria pygmaea	6.4*	0	0	1	3
Werneria sp2	0	0	0	0	7
Xenophyllum humile	5	0	0	0	0
Xyris subulata	0	4.5	1.9	1	0

Table S5. Mean and SD values for water chemistry, climate, disturbance, peat chemistry, and carbon for three different peatland vegetation types in the northern Andes. Homogenous groups after Tukey's HSD are denoted by superscript letters ($p < 0.05$), variables with non-significant differences in their means are n.s.

**Vegetation type**	**Cushion**	**Sedges**	**Grasses**	**True mosses**	**Sphagnum**

**Site conditions**					
Elevation	4384 ± 28.2	3569 ± 35.8	3693 ± 67	3359 ± 42.3	3170 ± 64.4
**Water chemistry**					
K^+ (mg l^{-1})	1.7 ± 0.364	0.45 ± 0.114	0.46 ± 0.097	0.91 ± 0.109	0.68 ± 0.153
Na^+ (mg l^{-1})	3.78 ± 0.969	1.16 ± 0.052	2.27 ± 0.448	1.09 ± 0.109	1.11 ± 0.094
Ca^{++} (mg l^{-1})	12.39 ± 2.356	7.11 ± 0.877	5.82 ± 0.465	8 ± 0.546	7.91 ± 0.774
Mg^{++} (mg l^{-1})	1.98 ± 0.511	0.15 ± 0.015	0.5 ± 0.216	0.38 ± 0.077	0.18 ± 0.02
pH	5.55 ± 0.158	4.84 ± 0.098	4.53 ± 0.185	5.04 ± 0.079	4.84 ± 0.1
Water electrical conductivity (μS cm^{-1})	145.6 ± 41.1	17 ± 1.8	33.7 ± 9.8	26.7 ± 4	20.7 ± 2.4
**Peat chemistry**					
Bulk density 0–10 cm deep (g cm^{-3})	0.06 ± 0.004	0.03 ± 0.004	0.06 ± 0.004	0.05 ± 0.005	0.05 ± 0.006
Bulk density 10–20 cm deep (g cm^{-3})	0.06 ± 0.005	0.05 ± 0.005	0.08 ± 0.01	0.06 ± 0.007	0.07 ± 0.007
Carbon content 0–10 cm (g m^{-2})	2382 ± 186	1397 ± 159	2041 ± 172	1707 ± 82	1445 ± 144
Carbon content 10–20 cm (g m^{-2})	2062 ± 130	1804 ± 179	2464 ± 244	1916 ± 195	2197 ± 229
Water storage 0–10 cm (% weight)	0.9 ± 0.009	0.93 ± 0.009	0.93 ± 0.006	0.92 ± 0.005	0.91 ± 0.009
Water storage 10–20 cm (% weight)	0.92 ± 0.007	0.94 ± 0.006	0.92 ± 0.011	0.91 ± 0.009	0.9 ± 0.012
Water table depth (cm)	14.62 ± 2.95	33.86 ± 2.97	15.59 ± 3.15	9.96 ± 2.08	16.26 ± 4.48
Mg content (g kg^{-1})	1.1 ± 0.293	0.42 ± 0.043	0.52 ± 0.085	0.49 ± 0.055	0.53 ± 0.073
K content (g kg^{-1})	2.91 ± 0.453	1.15 ± 0.219	0.84 ± 0.165	0.93 ± 0.134	2.24 ± 0.556
Ca content (g kg^{-1})	15.93 ± 1.37	19.01 ± 2.57	18.46 ± 2.45	13.37 ± 1.49	13.31 ± 1.92

Na content (g kg^{-1})	0.62 ± 0.104	0.54 ± 0.069	0.4 ± 0.046	0.3 ± 0.035	0.37 ± 0.046
